# Carbon-[*n*]Triangulenes and Sila-[*n*]Triangulenes: Which Are Planar?

**DOI:** 10.1021/acs.jpca.3c01820

**Published:** 2023-05-31

**Authors:** A. J. C. Varandas

**Affiliations:** † School of Physics and Physical Engineering, Qufu Normal University, Qufu 273165, P. R. China; ‡ Department of Physics, Universidade Federal do Espí rito Santo, 29075-910 Vitória, Brazil; § Department of Chemistry and Coimbra Chemistry Centre, University of Coimbra 3004-535 Coimbra, Portugal

## Abstract

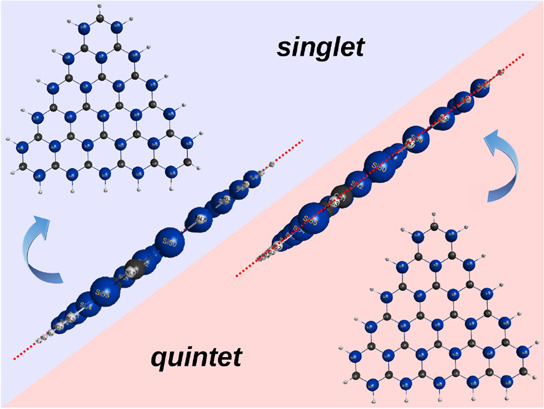

Using our recently suggested concept of a quasi-molecule (“tile”) and, in the case of the planarity here at stake, its generalization to larger than tetratomics, we explain why carbon [*n*]triangulenes tend to be planar, while hybrids, where just a few or even all *a*- or *b*-type carbon atoms are silicon-substituted (sila-[*n*]triangulenes), tend to be planar/nonplanar when compared with the unsubstituted carbon-[*n*]triangulenes. Because other spin states of the parent carbon- and sila-[*n*]triangulenes tend to correlate with the same tiles, it is conjectured that no structural changes are expected to depend on their spin state. Other polycyclic and sila-compounds are also discussed.

## 1 Introduction

The family of [*n*]triangulenes attracts much interest due to exhibiting magnetic properties and hence are potential components of molecular memory devices.^1−3^ While many nanographenes are closed-shell, the title ones have topologies that lead to open-shell electronic structures. Due to frustration with their conjugated networks, they assume high-spin states and show magnetic properties that attract attention. [*n*]Triangulenes also show interesting electronic and optical properties: their small band gaps make them semiconductors that absorb and emit light in the visible region of the spectrum. Overall, their unique magnetic and electronic properties make them promising candidates for applications in spintronics, electronics, and optoelectronics. Indeed, despite being reactive due to their open-shell nature, the development of such carbon-based nanostructures has been impressive: besides the popular [3]triangulene, [4]-, [5]-, and [7]triangulenes,^4−6^ as well as other appealing triangulene-based nanographenes, were on-surface fabricated.^7^

Although the properties of [*n*]triangulenes have been rationalized from electronic structure calculations at the level of Kohn–Sham (KS) density functional theory^8,9^ (DFT) and for *n* ≤ 4 using high-level ab initio calculations based on multireference theory,^10^ their planarity or nonplanarity is largely unexplained despite the impact on their attributes. For example, one expects the magnetic moments of the individual atoms to align more easily in a planar molecule, which creates a larger net magnetic moment for the molecule as a whole; when bent, they more likely cancel each other out, making the molecule less magnetic than when planar with the same atoms. By allowing for π conjugation, planarity also permits an efficient electronic delocalization, which enhances both the electronic stability and unique optical properties of the molecule. Coming with it may be a high degree of spin-polarization, which is of interest for spintronics. Still affected may be its magnetic anisotropy. Because this can be tuned by controlling the molecule orientation relative to an external magnetic field, planar molecules may be useful for applications in magnetic data storage. To sum up, judging the planarity of the title [*n*]triangulenes is key in providing information about their electronic structure and magnetic properties^1−3,11^ (and references therein). At the heart of this work, the planarity versus nonplanarity of carbon- and sila-[*n*]triangulenes is shown to be predicted from the quasi-molecules they embed.^12,13^

Being alternant hydrocarbons (not representable by Kekulé structures), [*n*]triangulenes form a bipartite lattice where all the nearest neighbors of one sublattice are members of the other sublattice: two types of carbon atoms (denoted *a* and *b*)^1−3^ are present, with type *a* alternating with the *b* carbon atoms. They are characterized^14−18^ by a high ground-state multiplicity, with a total spin *S* = |*n*_*a*_ – *n*_*b*_|/2, where *n*_*a*_ (*n*_*b*_) is the number of *a*- (*b*-type) C atoms. Such polycyclic aromatic hydrocarbons (PAHs) show zigzag edges and have the phenalenyl radical^19^ (or [2]triangulene, a unit fragment of a graphene sheet) as the series first member. With the main feature being the increase in the ground-state multiplicity with molecular size, their total spin is also given by *S* = (*n* – 1)/2, where *n* is the number of rows (or rings in one side of the triangle). So, their ground-state multiplicity is *n*.

From an X-ray study of the 2,5,8-tri*tert*-butylphenalenyl radical in the crystalline state,^20^ the phenalenyl fragment is known to have a slightly distorted *D*_3*h*_ symmetry. The next family member is [3]triangulene or Clar hydrocarbon,^21^ whose triplet state has been shown theoretically to have an energy of 20 kcal mol^–1^ lower than its singlet state.^22^ Indeed, plane-wave DFT calculations^23^ predicted triangulenes with high-spin ground states up to *n* = 15.

DFT calculations of nanographenes with zigzag edges (*n* = 2–5) and their silicon analogs were reported by Gapurenko et al.^1−3^ with the B3LYP functional and the 6-311++G** and 6-311+G** Pople-type basis sets. They identified stationary points on their potential energy surfaces (PESs), calculated for all possible spin states of each structure, and found them to prefer the spin state with the maximum possible value with energy gaps varying from 5 to 37 kcal mol^–1^. They also noted that they failed to locate symmetric structures of the low-spin states (mainly *C*_1_ systems were localized).^2^

DFT calculations have been efficient for geometry optimizations and, hence, are here also done for the title systems, mostly on their high-spin states at the unrestricted KS^9^ (UKS) level. Although suffering from some spin contamination, the mean values of the *S*^2^ operator show it to be moderate. Because the states of lower *S*_*z*_ are inherently multideterminantal, they are not directly accessible in the KS formalism. The usual practice is to perform broken spin-symmetry single-determinant calculations^3,24−29^ (and references therein). Basically, if the system has (*n* – 1) open shells or unpaired electrons, one first calculates the solution corresponding to *S*_*z*max_ = (*n* – 1)/2. Starting from the molecular orbitals (MOs) of this highest *S*_*z*_ solution, one then reverses the spin of one or two singly occupied MOs and optimizes the energy for the new spin distribution. The resulting energy is then usually assigned to that of an Ising Hamiltonian, i.e., the diagonal energy of a phenomenological Heisenberg Hamiltonian. The energy difference between the high-spin solution and these low-*S*_*z*_ solutions then leads to the effective exchange between unpaired electrons. Back to the Heisenberg Hamiltonian, the latter may last be used to estimate a more realistic spectrum of the system.^27^ Since we are not interested on the theoretical design of the title nanographenes, we resume to UKS DFT.

Isoelectronic symmetric triangulenes with silicon-substituted carbon atoms (here called sila-substituted^30^) along the perimeter of the [2]- and [3]-triangulenes have been synthesized as salts.^31−33^ Studies on sila-[*n*]triangulenes (*n* = 2–5) were also reported. Previously, sila-substituted systems with Si atoms replacing all the C atoms (i.e., Si_*m*^2^+4*m*+1_H_3*m*+3_, *m* = 2, 3, ...) were reported^34^ and found to be nonplanar. Likewise with hexasilabenzene, each ring was predicted to have a chair conformation.

Two variants of sila-substitutions in the triangulene framework were also considered. The first members of the families, where all the *a* or *b* carbon atoms are sila-substituted, were predicted to be planar^34^ and viewed as analogues of 1,3,5-trisilabenzene. Calculations were also reported for the systems . According to the latter,^2^ a planar structure is assumed whenever the substitution fills only all *b*-atom positions. Conversely, only the first two hybrid structures for [2]triangulene and [3]triangulene retained the highest *D*_3*h*_ symmetry when the substitutions occupied all *a*-atom positions: the symmetry was reported^2^ to be reduced for all other [*n*]triangulenes, with the planar *D*_3*h*_ structures of the sila-*a*-[4]triangulene and -[5]triangulene predicted as saddle points of indexes 2 and 3 [and destabilized relative to the corresponding minima by 9.2 and 9.5 kcal mol^–1^ without the vibrational zero-point-energy (ZPE) correction]. Such predictions are at the DFT level with the B3LYP functional, and hence, one wonders whether they afford sufficient realism. Indeed, any attempt to decipher planarity of a structure from a priori approximate calculations on the molecule itself is largely tautological.^12,13^

Left here aside is the fact that their experimental realization may be done in a medium at a finite temperature. This would require a prediction of their degree of flopiness and knowledge of whether they are in isolation or deposited on a surface. Clearly, accuracy would then be at demand, which would imply the use of a rigorous method and even extrapolation of the results to the complete basis set limit.^35−39^ Clearly beyond the goal of this work, the above issues will not invalidate the primary aim here of knowing whether the title systems are planar or nonplanar in isolation.

## 2 Basic Theory

### 2.1 Lemmas

We start by addressing the concept of quasi-tetratomic, and hence, reiterate Lemmas 1 and 2 reported elsewhere:^12^

**Lemma 1**: If all triads of points in a set are on a line, they are all on the same line (collinear in an Euclidean sense).

**Lemma 2**: If all tetrads of points in a set are on a plane, they are all on the same plane (coplanar in an Euclidean sense).

Because only planarity is at stake in the present work, let us consider four atoms (1234) that define a planar tetratomic molecule. Although the vectors *v⃗*_1_, *v⃗*_2_, *v⃗*_3_, and *v⃗*_4_ span a vector space *V*, there is a subspace *W* (*W* ⊂ *V*) that is a minimal spanning set. In fact, because all four vectors lie in a plane, only two (e.g., *v⃗*_1_, *v⃗*_2_) are enough to define any of the two remaining ones: the rank of the transformation matrix is 2. In terms of the atoms themselves, the proof of planarity may go as follows. First, a pair of tetrads (tiles) are formed such as to have three atoms in common (e.g., 1234 and 2345). Because three atoms define a plane and both tetrads share three atoms, all four atoms must lie on the same plane. Proceeding similarly and sequentially over all possible tetrads, if all the involved tiles are planar, then all atoms must share the same plane.^12^

### 2.2 Four-Atom Partitioning and Concept of Quasi-Tetratomic

Consider the fragmentation of a general PAH:
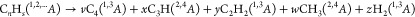
1where *n* = 4*v* + 3*x* + 2*y* + *w* and *s* = *x* + 2*y* + 3*w* + 2*z*. Note that only two electronic states of the fragments are indicated, and that one is typically interested in just one state of the parent molecule (singlet if closed shell, doublet when open shell, or an optimum spin state when noted). So, when focusing on the ground-singlet state of the parent, the radical species must combine in pairs. Although one or more electronic states are assigned along the text to a given molecular species, the reported calculations refer to the lowest of such states unless stated otherwise. Note further that the only nontetratomic fragment is H_2_. Because the quasi-tetratomics hope to mimic chemical-bound species and H_4_ is an extremely floppy van der Waals species, there is no great concern in using H_2_ (or 1/2H_4_, thus assuming without significant error that it is planar); all others are supposed to be at an equilibrium geometry (or stationary point; see later). If need be, the assumption may even be extended to 1/*m*H_2*m*_, with *m* being a small integer. When coming from competing sources, the H atoms are expected to arise from the weakest bonds where they are involved. However, because addition or removal of H atoms may play a key role in shaping a molecule, the use of such a strategy must be used with care or tested with the generalized quasi-molecule approach.^13^ Recall further that the C_3_H radical is planar in its ground-state cyclic geometry, hence more stable than in its isomeric linear form.^12,40^ Additionally, CH_3_ has a trigonal planar geometry^41^ (and references therein) with bond angles of 120°, although the energy cost for distortion to a pyramidal geometry is small. So, all fragments in eq 1 may be considered planar, with H_2_ typically assumed as playing no significant role as far as the valence geometry of the parent molecule is concerned. Even if repeatedly stated, if some clarification is required, use of the bisection strategy and generalized quasi-molecule concept^13^ is suggested.

Of course, the right-hand side in eq 1 must satisfy the atomic balance, with no atoms created or destroyed. Alternatively stated, eq 1 must obey the Wigner–Witmer correlation rules: only electronic states that combine to yield the singlet state of the parent benzenoid (in case of being a closed-shell species) can be involved. So, if *n* and *s* are even numbers, the parent molecule is assumed to be a singlet, which imposes restrictions on the number of open-shell fragments of a given type. Of course, higher spin states (triplet, quintet, and so on) may be allowed, as is the case in some of the systems considered in this work.

As indicated in eq 1, the Wigner–Witmer spin-spatial correlation rules allow for the formation of C_2_H_2_ in the singlet or triplet states, with a similar allowance for C_3_H, which may occur in its doublet or quartet states. By the same token, C_4_ may arise in its singlet or triplet states. In fact, C_4_ assumes nearly isoenergetic forms in its singlet and triplet forms,^42−44^ being fair to consider the above states as the most favorable ones. All occurrences depend on the energetically favorable path, once warranted the appropriate electronic state of the parent species. As noted above, even higher electronic states are possible, but unlikely to occur; this situation is therefore ignored unless noted otherwise.

Clearly, the products of a chemical reaction can only be identified when infinitely separated from each other. Still, one may conceptualize them to exist even prior to fragmentation, and we refer to them as quasi-molecules.^12,13^ Naturally, they cannot be assigned to a specific electronic spin state, but rather may occupy any of the states allowed by the spin-spatial rules. They are then virtual until dissociation occurs. Referred to as quasi-molecules, this is meant to allude to the concept of quasi-atom of Ruedenberg and co-workers,^45−48^ which was found most useful to understand the relative stabilities of the *trans*-bent, linear, and dibridged structures of Si_2_H_2_ and their C_2_H_2_ counterparts.^48^ Accordingly, the orbitals of the quasi-molecules may be viewed as distorted molecular basis set orbitals that are embedded in the actual molecular wave function of the parent molecule. Artificial in the quasi-molecule, they become the actual molecular orbitals once fragmentation occurs.

A further remark to note that the spin-spatial rules are valid irrespective of the form of the correlated species. Because minima and saddle points are both stationary points, one wonders to which type belongs a planar structure formed from a bissection (maxima are uninteresting stationary points and, hence, are here excluded). Minima represent stable species while saddle points are unstable relative to any push along a coordinate associated with an imaginary frequency. Hence, even if Lemma 2 implies a planar form, one cannot anticipate whether it is a minimum or a saddle point. This is to be expected, since the full characterization of a stationary point imposes a calculation of forces (hence, use of external, in the sense of additional, information) while Lemmas 1 and 2 involve only geometric (and energetic) arguments. Suffice it to recall that saddle points may act as minima if vibrational zero-point energy (ZPE) effects are brought into play.^49,50^ Of course, a saddle point implies (except if the potential energy surface is minimum-free) a minimum in its surrounding shape space. Deciphering its type may not be a problem once a planar structure is predicted from Lemma 2: a single-point calculation in a direction orthogonal to the plane of the molecule may be enough to clarify the problem. Indeed, for a large molecule, one expects the clarification to happen even during the subsequent bipartitions of the involved quasi-molecules.^13^ In any event, the approach remains much less expensive than optimization with calculation of forces.

As might be anticipated, the above strategy based on tiles (quasi-tetratomics) is cumbersome when dealing with molecules as large as the ones here considered without computational assistance. Prior to this, and to avoid the inherent combinatorial problem, we have suggested^13^ a simplified scheme based on the generalized quasi-molecule concept. It involves two steps: (a) split the parent molecule in two generalized quasi-molecules, ensuring that one is already a studied planar species; (b) if both fragments turn out to be planar molecules, continue the split process until the largest quasi-molecules are not larger than tetratomics. If the process is continued uninterruptly and found to yield nonplanar outcomes, the parent molecule is considered nonplanar. Otherwise, if only planar quasi-molecules are formed, the parent is considered planar. Suffice it to say that for this purpose there is one single path that yields planar tetratomic tiles, in agreement with Lemma 2.

### 2.3 Conjugation and Aromaticity: Does the Quasi-Molecule Concept Allow for Them?

We address the next three queries: (a) Is the quasimolecule concept reconcilable with the conjugation and aromaticity ones? (b) Since the latter are commonly invoked to imply planarity in cyclic aromatic molecules (and others), can the quasi-molecule concept meet the challenge? (c) Is it quantifiable? Such issues are examined in the remainder of this subsection.

A system of connected p-orbitals with delocalized electrons is called conjugated.^51^ In general, such a delocalization lowers the overall energy of the molecule and increases its stability. Commonly viewed to engage alternating single and multiple bonds, lone pairs may also be involved. Cyclic, acyclic, linear, or mixed structures may then benefit from it, with the benzene (C_6_H_6_) molecule being the most popular cyclic example. Also popular is the concept of aromaticity introduced by Hückel,^52^ who differentiated between aromatic and antiaromatic molecules. Both conjugation (also hyperconjugation^53^ or σ,π-conjugation^54^ or just orbital overlap;^55^ the literature is vast, with the reader being addressed to others through cross-referencing) and aromaticity lead to stabilizing interactions that influence the geometry, electron density, dissociation energy, or nuclear magnetic resonance properties among other physicochemical observables. Yet, the energetic criteria of aromaticity and antiaromaticity are based on assessments of energies relative to reference systems such as olefins or conjugated polyenes. So, despite their importance and widespread use, neither of these concepts has a strict physical definition and, hence, can be directly computed or measured. As a result, various auxiliary classifications emerged, with Grunenberg^56^ counting 45 different aromaticities in a tutorial review where the problem of quantification when dealing with ill-defined chemical concepts is addressed. Relatedly, Solá^57^ attributed what he called “the low reputation” of the aromaticity concept to the proliferation of its descriptors and advised the use of a set as large as possible to attain reliable conclusions. Hence, the current proposition is reckoned to deserve attention.

Conjugation and aromaticity are known to influence planarity in organic electronic materials.^58^ In fact, aromaticity was even deemed to be modified or controlled to represent a design opportunity.^58^ Although it is most popular in the study of traditional organic systems like benzene, coronene, etc., it is commonly used even in metal-bearing systems, where it is often called metallo-aromaticity.^59^ Recently, a shell (geometrical) model has been suggested that combines the role of aromaticity with topology and symmetry.^60−62^ By using a topological and electronic shell structure inherent in hexagonal PAHs, it has been shown^60^ how the celebrated Hückel and Clar rules^63,64^ for aromaticity and stability could be derived and connected to shell closures, with well-identified stability and aromaticity maxima versus number of π-electrons that resemble the ionization energy curves of the elements versus the atomic number. Despite the above and the fact that compounds said to be aromatic are generally flat, as they tend as such to possess extreme stability, cases exist^59^ where planarity does not mean higher aromaticity. For example, the minimum of the Al_13_^+^ structure (no imaginary frequency) has been predicted to be nonplanar, although it is more aromatic and shows a higher electron delocalization than two nearby planar geometries (saddle points). Indeed, the validity of different indicators of aromaticity has been explored in distorted benzene rings,^65^ with the authors led to conclude that there was not a single indicator that worked properly in all considered cases. Still, planarity has been shown to matter when studying the properties of gold clusters, with planar clusters found to be better electron acceptors than three-dimensional ones.^66^

Recapping, conjugation is possible by means of alternating single and double bonds in which each atom supplies a p orbital perpendicular to the plane of the molecule. The question then emerges: can the quasi-molecule concept allow to explain cyclic aromatic molecules where conjugation occurs? The tempting answer from the systems we have studied thus far^12,13^ is a clear yes. For example, we have shown that it explains the planarity of benzene, while octatetraene is predicted to be nonplanar. Recall in this regard that C_3_H, a pilar tile, is among the simplest molecules where conjugation can occur since it has three contiguous carbon atoms, where each supplies a p orbital perpendicular to the molecular plane. Moreover, it helped to explain the planarity of a variety of PAHs,^12,13^ and the reader may appreciate in this work that it continues to be helpful in explaing planarity for [*n*]triangulenes and sila-[*n*]triangulenes.

One wonders at this point whether the quasi-molecule concept needs to be quantifiable. The answer is negative if one recalls that there are many fruitful concepts in chemistry that are not so.^56,57^ Still, we may conjecture about using statistics to define an index of planarity likelihood, a topic that will not be pursued in this work. Suffice it to emphasize that planes are well-known geometric primitives, the fundamental nature of which is easily perceived by recalling that slices of a 3D object are used as proxies for creating shape abstractions in art and engineering.^67^ In fact, it was the key nature of planarity that posed the motivation for this work jointly with the asset that triangulenes possess unique magnetic properties (hence, are of great interest in materials science^1,68^). Indeed, it is not clear which [*n*]triangulenes and sila-[*n*]]triangulenes are planar among the numerous members of their families. Besides being challenging and offering an application of the above Lemma, the approach allows for significant computational savings. To our understanding, the present results are as encouraging as in past applications.

### 2.4 Electronic Structure Calculations

All results here reported employed the MOLPRO package^69^ for electronic structure calculations. In all cases, single-reference (SR) methods are utilized, without imposing symmetry.^70^ Eventually feasible at higher levels of theory on a single-system basis (e.g., employing CBS extrapolated conventional^35,36^ or explicitly correlated methods^37−39^), no attempt has been deemed worth pursuing at this occasion, but rather the most commonly used SR approaches are employed. For the quasi-tetratomics (tiles), a high accuracy is deemed to be required, since they should mimic their rigorous Born–Oppenheimer solution. Even so, the sort of accuracy previously reported^12^ is judged enough. In fact, even if explicitly correlated SR methods would occasionally be possible, the ones actually employed should be enough for the endeavor. More important, perhaps, one should recall that the high reactivity of the PAHs is commonly associated with a substantial radical character, thus requiring multireference methods for a proper description of the electronic wave function.^10,71^ This is prohibitive for frequency calculations and, hence, not attempted: the aim is to provide insight into the predictions here reported (irrespective of size) rather than give a state-of-the-art description of the PAH electronic properties.

So, the highest employed level of theory uses the canonical coupled cluster singles and doubles method, including perturbative triples, CCSD(T), commonly viewed as the gold standard of computational chemistry. When affordable, the correlation consistent triple-ζ basis sets of the Dunning family are employed:^72,73^ cc-pV*X*Z (simply V*X*Z) or, when affordable, their augmented variants aug-cc-pVTZ (AV*T*Z). When involving silicon, the corresponding V(*X* + *d*)Z and AV(*X* + *d*)Z basis sets are utilized.^73−75^ For medium to large molecules, or prospective calculations, we employ UKS DFT^9^ with the recommended AV*T*Z basis sets. Specifically, the popular B3LYP^76,77^ and M06-2X^78,79^ functionals are employed with the recommended AV*X*Z basis sets. When unaffordable, or the burden is too heavy otherwise, V*X*Z basis sets^72,73^ are utilized occasionally at the V*D*Z level. In extreme cases, to simplify the computational cost, DFT/B3LYP and DFT/M06-2X calculations with Huzinaga’s^80^ MINI basis sets are employed.

A parenthetical point is already noted elsewhere.^12^ Doubts have been raised in the literature about predictions based on the current basis sets due to potential linear dependencies.^81,82^ Since any pruning of the basis sets to reduce such dependencies is, to our knowledge, not possible with MOLPRO, calculations employing atomic natural orbital basis sets of V*X*Z quality are occasionally reported as recommended by Martin and co-workers.^83^ Specifically employed are the ANO-VT-*X*Z basis sets^84^ (restricted to double- or triple-ζ), where the exponents of the primitive Gaussian functions were subject to uniform scaling to ensure satisfaction of the virial theorem for the atoms.

The same levels of theory are employed for the optimizations and force calculations. Except where indicated, all utilized the default convergence criteria of MOLPRO. Hence, minute deviations from eventually symmetrical structures should rightfully be seen as reflecting nothing but the attained numerical accuracy. In pratice, deviations of a tenth of a degree in angles are likely to have no numerical significance, while bond distances tend to be reliable up to three (perhaps four) numerical decimals; for convenience, four decimals are always indicated. Like previous work on this topic, only the valence electrons are correlated.

Suffice it to add that relative energies are given for the best method and basis set; for reference, the total energies are often quoted. No attempt is made to fully describe specific electronic states with unpaired electrons. Still, unrestricted DFT, which is often viewed as accounting somehow for the static correlation, seems to confirm the high-spin multiplicity of the ground state of the title PAHs and their sila-analogues. Indeed, rather than use for theoretical design,^1,2,27,85^ their aim here is mostly for probing the predictions from the quasi-molecule theory. Of course, small deviations from planarity may be argued to be irrelevant when recalling that some floppiness may be of no effect when studying such molecules deposited on a surface. Even so, we qualify as nonplanar the cases where all atoms are not in the same plane. Having in mind the modest realism of some of the calculations, attention is seldom paid to quantify the deviations from planarity. When slight, the molecule is considered quasiplanar, as indicated by a thin (rather than boldface) *P*, otherwise a *N* qualifies.

## 3 Results and Discussion

In an attempt to provide even coverage, we consider first in section 3.1 the carbon-[*n*]triangulenes that are preliminarily addressed elsewhere.^13^ Then, previous to larger silicon-substitutions, we examine in section 3.2 the simplest sila-[*n*]triangulene member cases. The extension to sila-[*n*]triangulenes for up to *n* = 5 appears in section 3.3, and a brief detour on other sila-[*n*]triangulenes with all-*a*- and all-*b*-carbon-atom substitutions is in section 3.4. Exploratory work on other spin states is discussed in section 3.5.

### 3.1 Carbon [*n*]Triangulenes

Carbon [*n*]triangulenes have been reported up to *n* = 7,^6^ although we consider them here only up to *n* = 5.

The generalized quasi-molecule strategy then gives
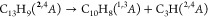
2
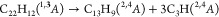
3
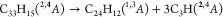
4
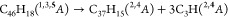
5

**Figure 1:**
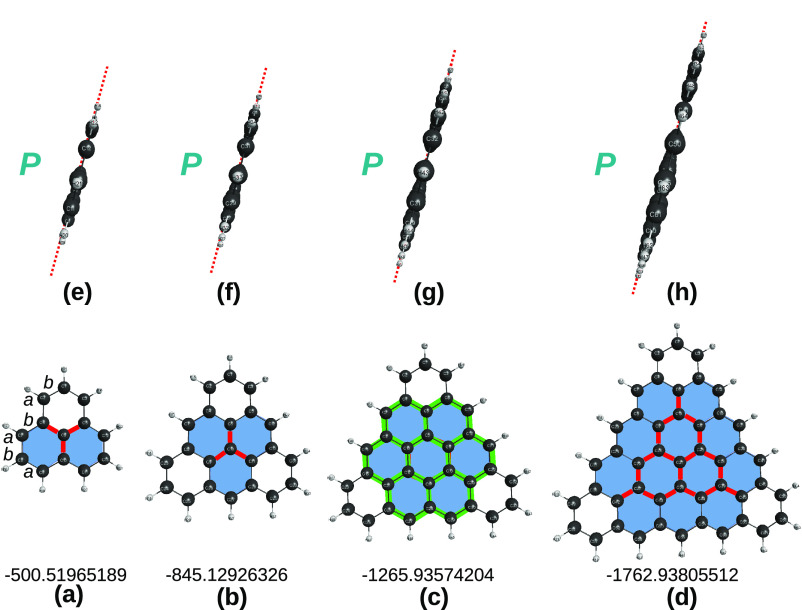
Optimized structures of carbon [*n*]triangulenes (*n* = 2–5) at the DFT level of theory with the B3LYP functional and V*D*Z basis set. Indicated is the calculated total energy (given for reference as reported in the MOLPRO output) and, in blue, the essentially perfect planar (*P*) shape of the optimized structures. Also indicated is the notations *a* and *b* used to label the carbon sites. Furthermore, shown in color are various carbon clusters that are generalized quasi-molecules previously used^13^ to assess the planarity of the corresponding [*n*]triangulenes: C_4_ in (a) and (b), C_24_ in (c), and C_16_ in (d). In this and the following figures, distances are in Å, angles are in deg, and total energies in *E*_*h*_.

As seen from eqs 2–5, all spin states of the parent molecules correlate with the ones of the (generalized)-tiles, irrespective of the parent spin-state. Since, to our knowledge, no experimental details are known in the gas-phase molecules, we confront our predictions with the results of DFT calculations, the majority at the V*D*Z basis set level. The results from these tightly converged (5.0 × 10^–6^*E*_*h*_ Å^–1^) calculations for the [*n* ≤ 5]triangulenes are in Figure 1. A key point refers to the spin state of C_3_H. While it can be both the doublet and the quartet to obtain the optimum quartet spin of C_33_H_15_(^2,**4**^*A*), it is mostly the quartet [as indicated by the bold superscript in eq 5] in the case of C_46_H_18_(^1,3,**5**^*A*).

**Figure 2:**
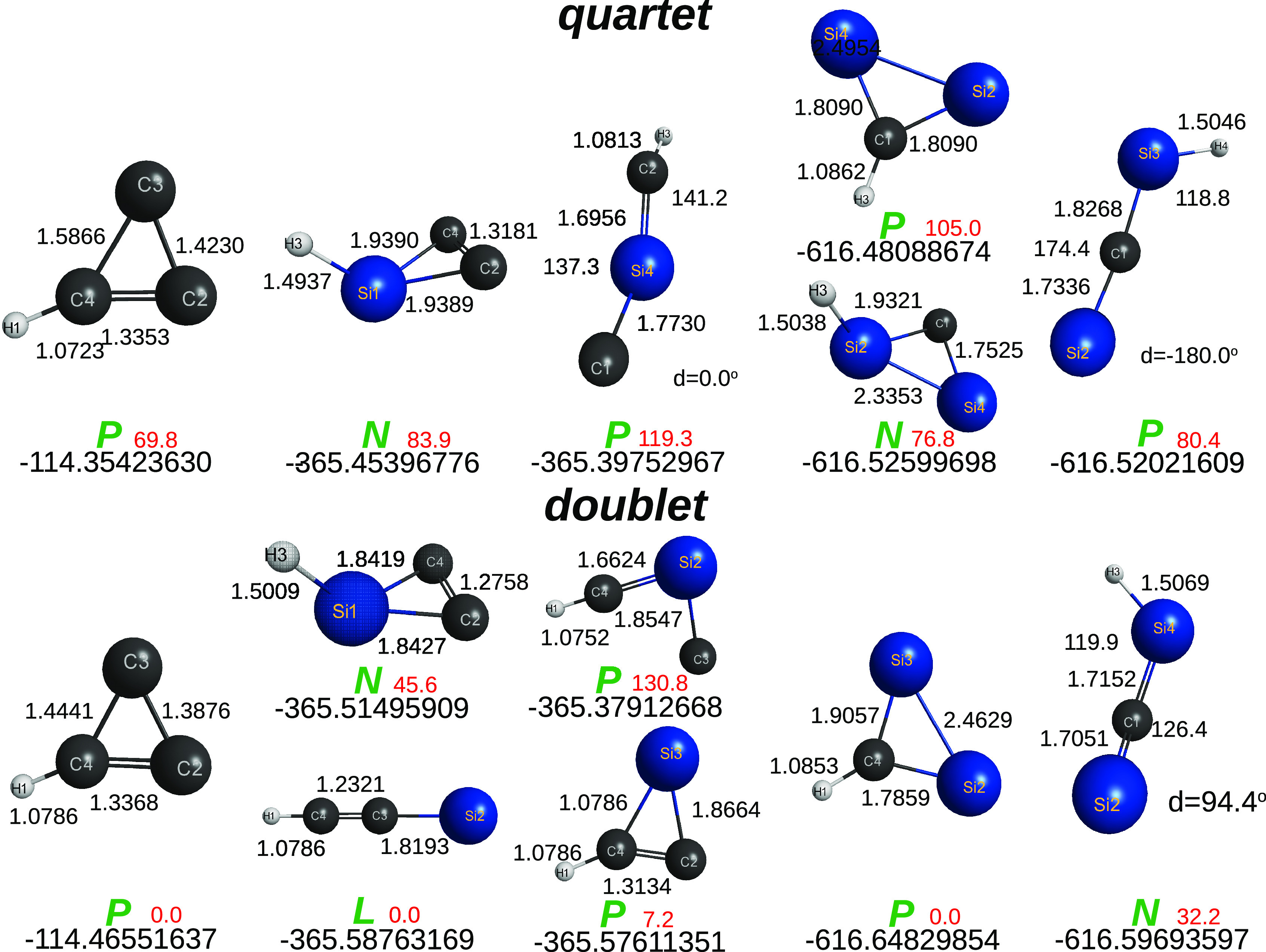
Optimized structures at the CCSD(T)/AV(*X* + *d*)Z level of theory of the relevant quasi-tetratomics (tiles) employed for the interpretation of the carbon and sila-[*n*]triangulenes. Symbols as in Figure 1. Given in red (in kcal mol^–1^) after the P or N symbols are the energies relative to the isomer with the lowest energy. See also refs (12 and 13).

Figure 2 shows the optimized tiles at the CCSD(T)/AV*T*Z level of theory. Note that the HC_3_ tiles are planar both in the doublet and quartet spin states, and hence, we expect that this issue has no implications in the predictions made for the carbon [*n*]triangulenes, irrespective of *n*. Note further that the larger generalized quasi-molecules, C_24_H_12_(^1,3^*A*) and C_37_H_15_(^2,4^*A*), can be either in their singlet and triplet or doublet and quartet spin-states, hence, it is assumed to be their lowest spin states; see the first four entries of Table 1. Although not relevant for the carbon [*n*]triangulenes, this is not so for the sila hybrids. Indeed, this is the key point in predicting their planarity, as shown later in this work.

**Table 1:** Bipartitions of C_9_H_13_, C_22_H_12_, C_33_H_15_, C_46_H_18_, and Their Sila-All-*a*- and Sila-All-*b*-[*n*]Triangulene Derivatives^a^

*n*-/*a*,*b*	quasi-molecules	obs.	quasi-molecules	obs.	quasi-molecules^b^	obs.	quasi-molecules^b^	obs.
2	C_3_H + C_10_H_8_	*P*	C_6_H_6_ + C_4_H_2_	*P*		*P*		
3	3C_3_H + C_13_H_9_	*P*	C_3_H + C_10_H_8_	*P*	C_6_H_6_ + C_4_H_2_	*P*		
4	3C_3_H + C_24_H_12_	*P*	C_6_H_6_ + C_18_H_6_	*P*	6C_3_H	*P*		
5	3C_3_H + C_37_H_15_	*P*	C_13_H_9_ + C_24_H_6_	*P*	C_6_ + C_18_H_6_	*P*	C_6_ + 6C_3_H	*P*
2-*a*	3CSi_2_H + C_3_Si	*P*						
3-*a*	3CSi_2_H + C_7_Si_6_H_9_	*P*	3SiC_2_H + CSi_3_	*P*				
4-*a*	3CSi_2_H(^**2,4**^A) + C_12_Si_12_H_12_	*N*						
5-*a*	3CSi_2_H(^2,**4**^A) + C_18_Si_19_H_15_	*P*	C_6_Si_7_H_7_ + C_12_Si_12_H_8_	*P*	C_3_Si_3_H_2_ + C_9_Si_9_H_6_	*P*	3C_3_Si_3_H_2_	*P*
2-*b*	3SiC_2_H + CSi_3_	*P*						
3-*b*	3SiC_2_H + C_6_Si_7_H_9_	*P*	3Si_2_CH + SiC_3_	*P*				
4-*b*	3SiC_2_H(^**2,4**^A) + C_12_Si_12_H_12_	*P*	C_3_Si_3_H_6_ + C_9_Si_9_H_6_	*P*	3C_3_Si_3_H_2_	*P*		
5-*b*	3SiC_2_H(^2,**4**^*A*) + C_19_Si_18_H_15_	*P*	C_7_Si_6_H_7_ + C_12_Si_12_H_8_	*P*	C_3_Si_3_H_2_ + C_9_Si_9_H_6_	*P*	3C_3_Si_3_H_2_	*P*

a Except where indicated otherwise (the optimum spin state is in bold), the tetratomic tiles are considered in their lowest spin states.

b No attempt is made to include all quasi-molecules resulting from the possible bipartitions, numbered first, second, etc., from the left to right columns. See the text.

### 3.2 Hybrid [2]Triangulenes: 1-, 2-, 3-, and 4-Atom Sila-Substitutions

**Figure 3:**
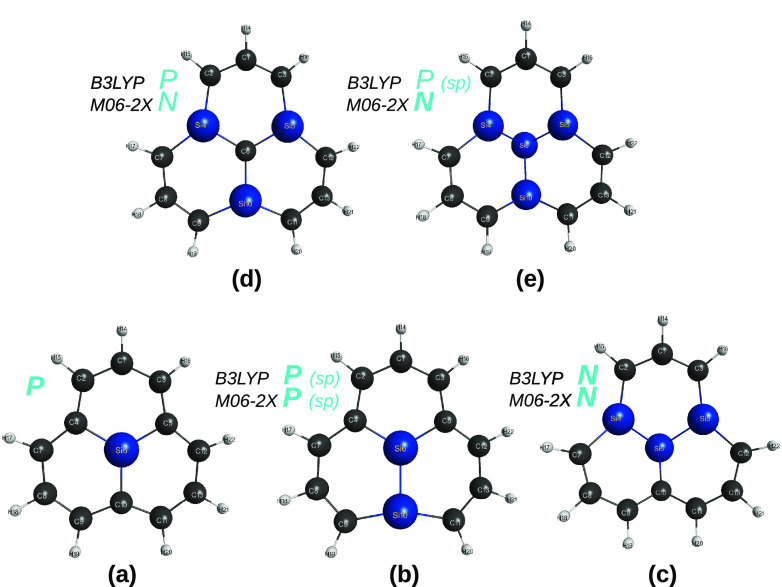
Optimized structures of five sila-[2]triangulenes, C_13–*n*_Si_*n*_H_13_ (*n* = 1–4), at DFT level of theory with the B3LYP and M06-2X functionals and the MINI and V*D*Z basis sets. Indicated in blue is the planar (*P*) vs nonplanar (*N*) shape of the optimized structure. Structures predicted to be a saddle point are indicated as “sp”.

**Figure 4:**
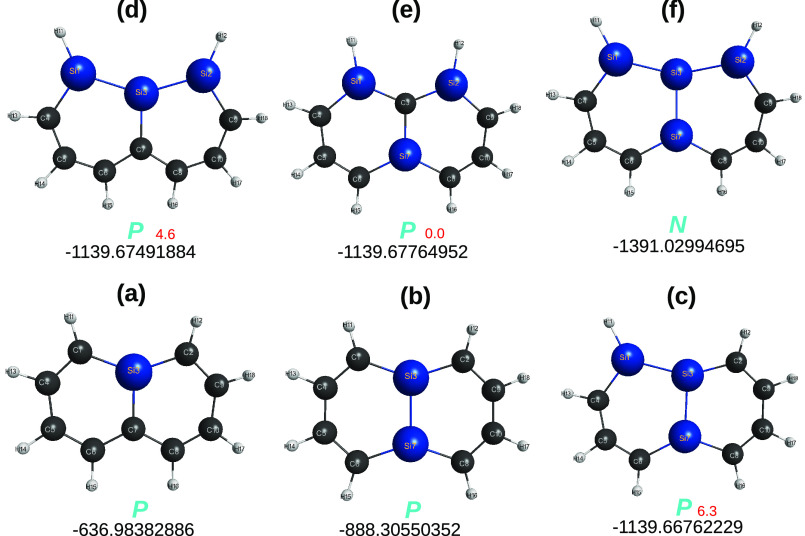
Optimized structures of C_10–*n*_Si_*n*_H_8_ (*n* ≤ 4) at the DFT/B3LYP/V*D*Z level of theory. Given in red (in kcal mol^–1^) after the *P* symbol for the three-silicon-atom molecules are the energies relative to the isomer with the lowest energy in panel (e). Symbols as in Figure 3.

**Figure 5:**
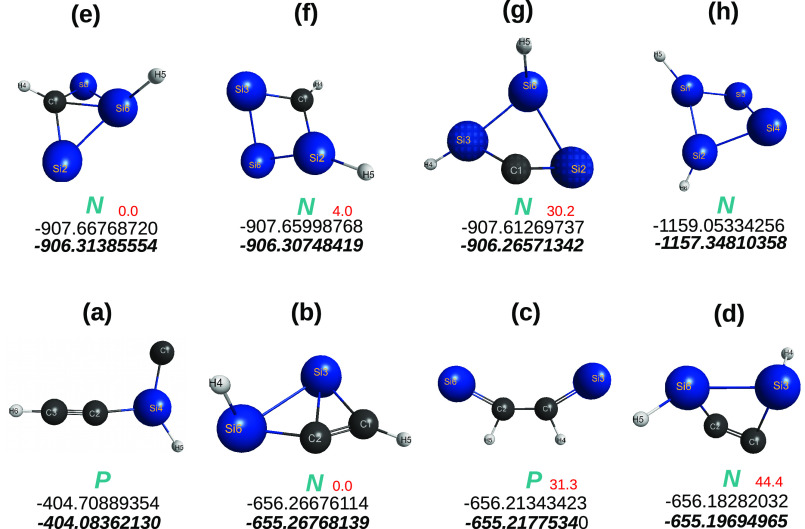
Optimized C_4–*n*_Si_*n*_H_2_ (*n* ≤ 4) structures at the CCSD(T)/AV*T*Z level of theory. The top energies are for DFT/M06-2X/AV*T*Z, and the bottom ones for CCSD(T)/V(*T* + *d*)Z. Given in red (in kcal mol^–1^) after the *P* and *N* symbols are the CCSD(T) energies relative to the corresponding lowest energy isomers. Symbols as in Figure 3.

**Figure 6:**
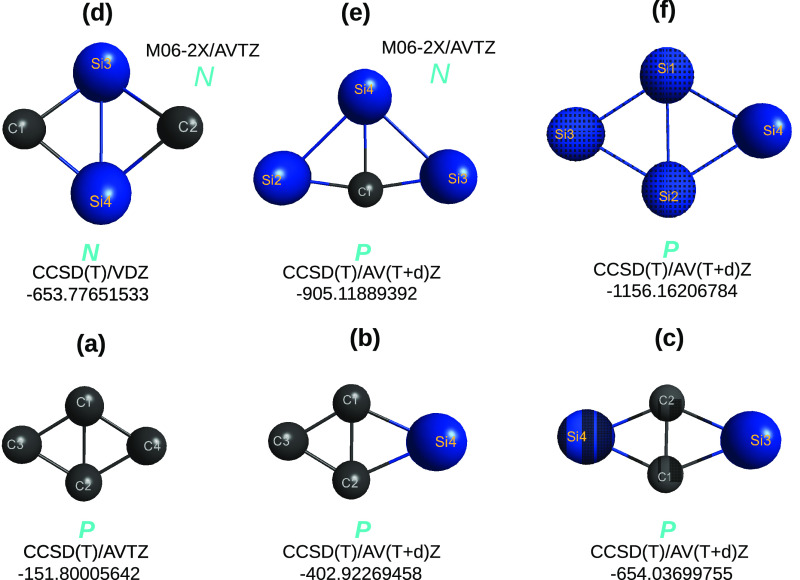
Optimized C_4–*n*_Si_*n*_ (*n* ≤ 4) structures at the CCSD(T) level of theory with the indicated basis sets. Symbols as in Figure 3.

The generalized quasi-molecule strategy is here used for studying the [2]triangulene. The following partitions apply:
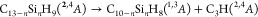
6
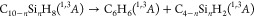
7
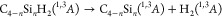
8with *n* ≤ 4 in the above equations. The whole process is shown in Figures 3–6. The first of these, for various sila-[2]triangulenes, shows the results of DFT calculations with both the B3LYP and M06-2X functionals and V*D*Z basis sets. Any attempt to go beyond this level of theory is too expensive with the computational means at our disposal, although calculations were also done for hybrids (b), (c), and (d) with the ANO-VT-V*T*Z basis sets.^84^ As shown in Figure 4, only one hybrid of the sila-naphthalene is found to be nonplanar, which discards the possibility of the sila-[2]triangulene in panel (e) of Figure 3 to be considered planar. The split-process continues as in eq 7, where benzene is the quasi-molecule commonly known and shown elsewhere^12^ to be planar.

The quasi-molecules outcoming from the split in eq 7 are shown in Figure 5. Clearly, no attempt has been made to gather all possible minima, a task well beyond the goal of the present work. Of such optimized structures, only the ones shown in panels (a) and (c) turn out to be planar, thus eliminating via panels (b) and (d) to (f) of Figure 5 any possibility for the parent sila-[2]triangulenes to be planar. For example, the C_2_Si_2_H_2_ in Figure 5 can hardly support that panel (b) of Figure 3 is planar, since its isomers in panels (b) and (d) of Figure 5 are nonplanar. In fact, the sila-[2]triangulene in panel (b) of Figure 3 is predicted to be a planar saddle-point both at B3LYP/V*D*Z and M06-2X/V*D*Z levels of KS DFT, thus letting one anticipate that the minimum structure is nonplanar, which is corroborated by the imaginary vibrational frequency that corresponds in both cases to the same out-of-plane vibrational mode.

A further remark to note is that 2 of the 3 dihedrals that define the molecule of Figure 3a correspond to perfect planarity, while the third referring to the (4,2,3,6) tetrad deviates by 3.4°; for whatever reason, such a dihedral can only be of perfect planarity, since both (3,2,1,4) and (1,2,4,5) tetrads share three atoms with each other; hence, all five atoms must be coplanar. Because the tetrad (4,2,3,6) shares three of the above five atoms, all six atoms must share the same plane.^12^ We therefore attribute such a small deviation of the planarity to the methodology and possible numerical inaccuracies.

The final step translates into eq 8 and Figure 6, which shows the involved C_4–*n*_Si_*n*_(^1,3^*A*) structures. To summarize, only the sila-[2]triangulene in panel (a) of Figure 3 is predicted to be planar, which corroborates the actual DFT results here with both the B3LYP and M06-2X functionals.

**Figure 7:**
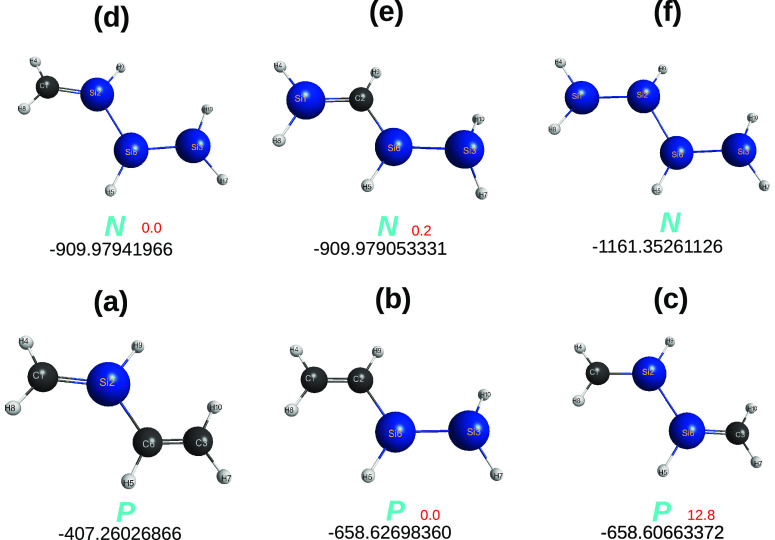
Optimized C_4–*n*_Si_*n*_H_6_ structures at CCSD(T)/AV*T*Z level of theory. Symbols as in Figure 3. Given in red (in kcal mol^–1^) after the *P* and *N* symbols are the energies relative to the lowest energy isomer.

One wonders at this stage whether a different split process could yield a distinct prediction. In an attempt to provide further evidence, we considered the two-step process:
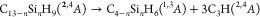
9
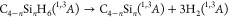
10with *n* ≤ 4 in these equations. As shown in Figure 7, the calculated results support the above conclusions.

### 3.3 Sila-[*n*]Triangulenes: All-*a*- and All-*b*-Carbon-Atom Substitutions

We consider next the sila-[*n*]triangulenes previously studied by Gapurenko et al.,^1−3^ where all *a* or *b* carbon atoms that reflect the sublattice balance and symmetry^62^ have been replaced by silicon atoms. Our predictions will be compared with DFT optimizations using either AV(*D* + *d*)Z basis sets (for the first member of the [*n*]triangulene family) or simpler wave functions down to the Huzinaga’s^80^ MINI ones.

**Figure 8:**
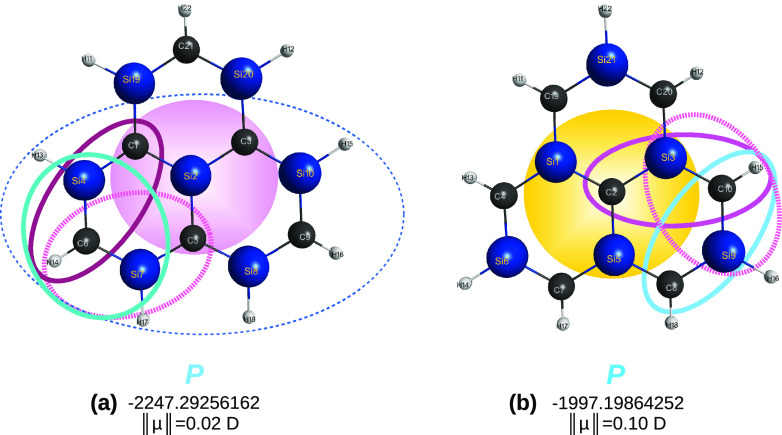
Sila-all-*a*- and sila-all-*b*-[2]triangulenes studied in the present work at the DFT level of theory with the B3LYP and M06-2X functionals and MINI, AV(*D* + *d*), and ANO-VT-V*T*Z basis sets. The structures shown refer to the simplest B3LYP/MINI calculations. They are essentially planar, except for small (typically ≲ 1°) deviations in panel (a), the dihedral angles involving the atoms (3,2,1,4), (1,4,6,7), and (4,1,11,19); panel (b), the dihedral angles (3,2,1,4), (1,4,6,7), and (2,5,8,9).

Figure 8 illustrates the cases of sila-*a*-[2]triangulene and sila-*b*-[2]triangulene, in panels (a) and (b), respectively. In panel (a), the split is first done as indicated in eq 6 by calling attention for the fact that the substituted naphthalene generalized quasi-molecule (within the large dashed ellipse in dark blue) has been shown to be perfectly planar (which has been here corroborated through DFT calculations employing both the B3LYP and M06-2X functionals and V*D*Z basis sets), while the two other smaller ellipses in light blue and brown indicate that all atoms are in the same plane since they share three atoms of the associated tiles in common with each other. A similar approach is adopted to imply the planarity of sila-*b*-[2]triangulene: the ellipses share with each other three atoms of the involved tiles.

**Figure 9:**
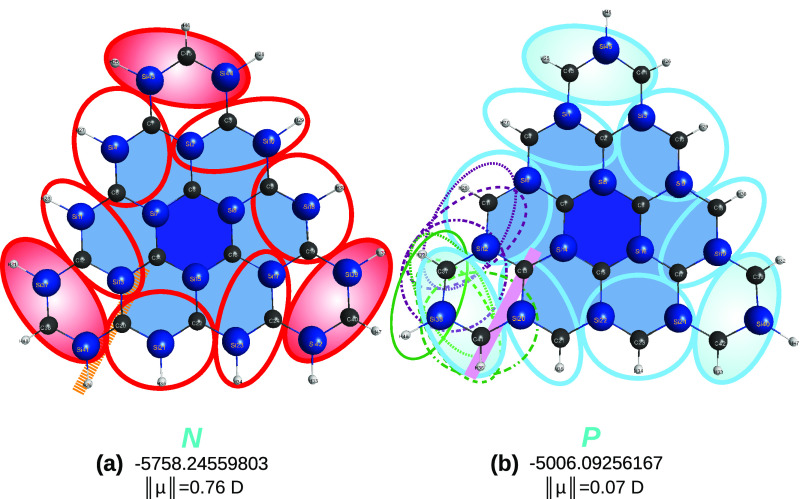
Sila-all-*a*- and sila-all-*b*-[4]triangulenes. From the naked eye, both look planar. However, the former, in panel (a), shows some dihedral angles deviating significantly from planarity, which suggests a *N* classification, at least of weakly nonplanar. Instead, the sila-all-*b*-[4]triangulene can largely be considered as planar, in agreement with the predictions at the levels of theory here afforded.

Because the sila-*a*- and sila-*b*-[3]triangulenes are not expected to show features distinct from the sila-[2]triangulenes, we move upward in size to show in Figure 9 the optimized structures of the sila-*a*- and sila-*b*-[4]triangulenes. They have been optimized with both the B3LYP and M06-2X functionals and the MINI basis sets. The results, shown for the M06-2X functional, are self-explanatory, simply by counting the number of planar (in light blue) and nonplanar (red) tiles. Indeed, most dihedral angles in case (a) show values that differ significantly in absolute value [up to 60° for the tetrad (19,9,8,30)] from 0 or 180°, whereas in case (b), only seven do so and by less than 1.8°. This is corroborated by the calculated dipole moments at the equilibrium geometry: it has a norm of 0.76*D* for sila-*a* [4]triangulene, while for the sila-*b* congener it is about 10× smaller. To show that all atoms share the same plane in panel (b), it is sufficient to note the brownish panels (they may be continued rightwards by the greenish ellipses, then upward, etc until closing the triangle) and see that they all share three atoms in common with the previous one, thus warranting that all are in the same plane. To summarize, the sila-*b*-[4]triangulene is predicted to be planar, in contrast with the sila-*a*-[4]triangulene, which is predicted to be nonplanar (*N*).

To investigate whether the coronene atoms (in the shaded blue area of Figure 9) and the ones of the peripheral C_3_H tiles are on the same plane, one may use the fact that the ground-doublet C_3_H has a linear isomer (a full account on linear versus nonlinear C_*n*_H species up to *n* = 8 will be given elsewhere). In fact, calculations at the B3LYP/AV*T*Z level predict the ground-doublet HCCSi to be linear, while the ground doublet HCSiC is planar (L-shaped) with a total energy of −365.93771770 *E*_*h*_, hence 114.4 kcal mol^–1^ above the former. Regarding HCCSi, only the minimum linear structure (*E* = −366.15032447 *E*_*h*_) is predicted at the M06-2X/AV*T*Z level, with HCSiC being a saddle point located above by 128.9 kcal mol^–1^. At the restricted CCSD(T)/AV(*T* + *d*)Z level, the results for HCCSi and HCSiC are −365.58763169 *E*_*h*_ and −365.57611346 *E*_*h*_, respectively; the latter corresponds to a cyclic planar structure located 7.2 kcal mol^–1^ above the former, but cannot be confidently related to the above ones prior to further study. We have also performed calculations at the UCCSD(T)/AV(*T* + *d*)Z level, which predict HCCSi to be linear and CSiCH planar L-shaped minima, respectively. Similar to the B3LYP prediction, the total energies are −365.50203212 *E_h_* and *E* = −365.37911979 *E_h_*, in the same order, hence, separated by 77.1 kcal mol^–1^. Clearly, the large energetic differences may be attributed to the distinct methods. Suffice it to add that both the DFT and the CCSD(T) methods have been utilized in the unrestricted variant, thus hoping to account in both cases for some static correlation within the corresponding SR approaches.

**Figure 10:**
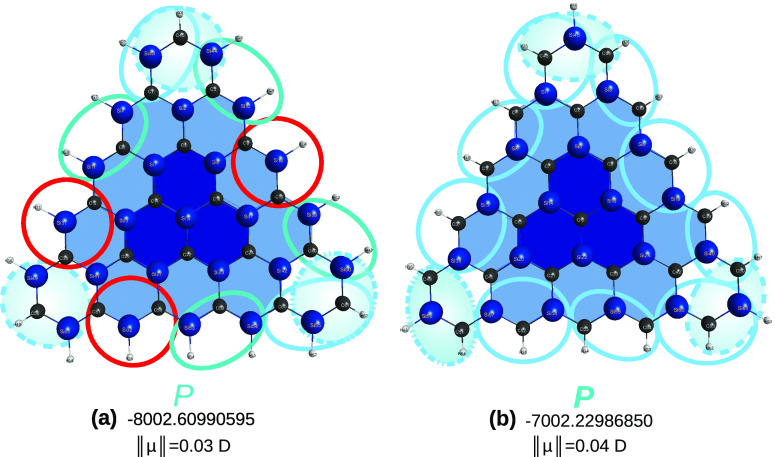
Sila-all-*a*- and sila-all-*b*-[5]triangulenes. Symbols as in Figure 9.

Regarding the sila-[5]triangulenes, they have been reported as saddle points of index 4 at DFT/B3LYP/6-311+G** level of theory in ref (2). In the present work, we have carried out a fresh DFT/M06-2X/MINI calculation that, despite the smallness of the basis sets, already took 3 days of uninterrupted computational work each to compute the harmonic vibrational frequencies. In an attempt to warrant a proper characterization of the stationary point, a tight convergence of 5 × 10^–6^*E_h_* Å^–1^ was imposed. For the sila-*a*-[5]triangulene, the prediction was a planar minimum structure. In accordance with its near-planar geometry, a dipole minimum with a small norm of 0.03 D was obtained; see Figure 10. Indeed, only two dihedral angles of 0.9° and 6.2° were observed involving the atom tetrads (19,9,18,30) and (12,36,37,38), respectively. Yet, out of the 186 vibrational frequencies that characterize the predicted minimum structure, 8 turned out to be low vibrations: (13.7, 16.0, 18.3, 34.2, 41.4, 42.0, 48.5, and 49.5 cm^–1^). In turn, for the sila-*b*-[5]triangulene, the prediction was a perfectly planar minimum structure, except for one dihedral angle involving the tetrad (12,36,37,38) that deviates by ∼2.5°, and four low vibrations that show values of 17, 18, 20, and 47 cm^–1^. Given the smallness of the basis set here employed, it is impossible to state that the current prediction is drastically distinct from the ones previously reported^2^ (apparently, only for the sila-*a* hybrids) from B3LYP/6-311+G**, except for being predicted to be minima. To explore further the role of convergence, we have redone the optimization of the sila-*a*-[5]triangulene with the convergence tightened to 10^–6^*E_h_* Å^–1^. This merely confirmed the above results, except for eliminating the deviation in the dihedral of the tetrad (19,9,18,30) while enhancing slightly the one of (12,36,37,38) to 6.8°. In summary, both the sila-*a*-[5] and sila-*b* congeners are here catalogued as planar, according to the larger number of encircled tiles in light blue. Of course, one must recall that this refers to the quintet spin state of the sila-[5]triangulenes, and hence, a fair comparison with experimental data should not only employ larger basis sets, but account for other (nonoptimal) spin multiplicities, a point that is readdressed briefly later.

The simplest way to quest planarity of the sila-[5]triangulenes is by considering the following splits:
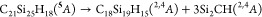
11
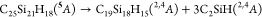
12

Since DFT calculations here performed with the B3LYP and M06-2X functionals and MINI basis sets predict both C_18_Si_19_H_15_(^2^*A*) and C_19_Si_18_H_15_(^2^A) to be perfectly planar (no single dihedral deviates from planarity for both the doublet and quartet spin states), it is reasonable to anticipate potential deviations from planarity through the Si_2_CH(^2,4^A) and C_2_SiH(^2,4^A) [to be read in what follows CHSiC(^2,4^A)] tiles. As shown (by the bluish-filled tiles in Figure 10, circled in dashed blue), the three tiles in both triangulenes can be arranged such as to involve planar tiles, thus corroborating their high degree of planarity.

**Figure 11:**
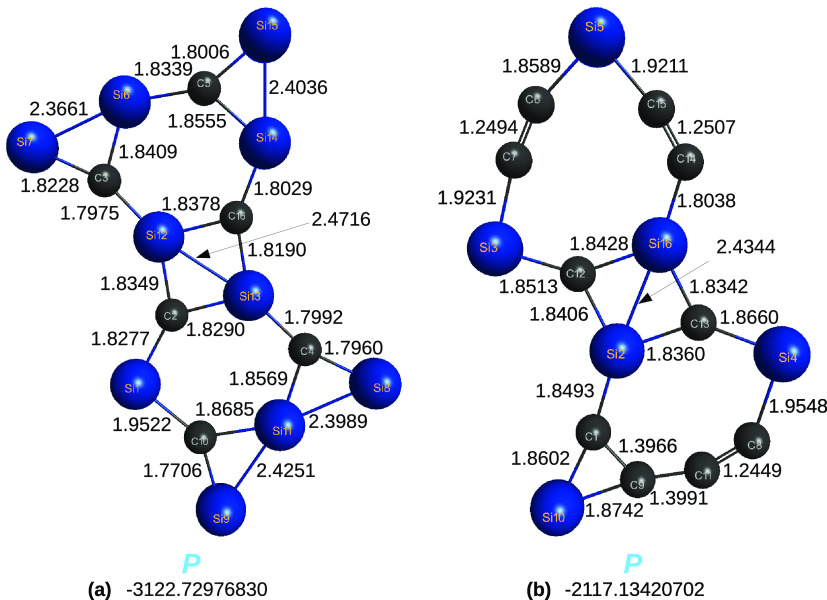
Optimized structures of C_6_Si_10_ and C_10_Si_6_ hybrid C/Si clusters at DFT/M06-2X/ANO-VT-*D*Z level of theory.

One may wonder about the properties of the C_6_Si_10_ and C_10_Si_6_ internal atomic clusters ineqs 13 and 14, respectively, and panels (a) and (b) of Figure 11. As in previous calculations, which predicted C_16_ to be planar, corresponding fresh calculations here performed for C_6_Si_10_ and C_10_Si_6_ led to similar results: the former is nearly perfectly planar [a single dihedral angle involving the tetrad (2,3,5,15) deviates by 2.7°; see panel (a) of Figure 11], while the latter in panel (b) of Figure 11 shows perfect planarity. Since we are unaware of such structures having been previously reported, we have redone their optimizations at the M06-2X/ANO-VT-DF level of theory and report their geometries in panels (a) and (b) of Figure 11; for the Cartesian coordinates and harmonic vibrational frequencies, see the Supporting Information (SI).

The above suggests that an analysis similar to the one in the previous paragraph can be based on the following splits:
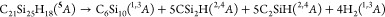
13
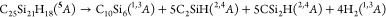
14

Indeed, this yields the same predictions as above. It should be stated that no attempt has been made in the present work to determine the structures of other potential isomers of the 16-atom C–Si clusters. Additionally, the 4 hydrogen molecules were assumed to play no fundamental role.

**Figure 12:**
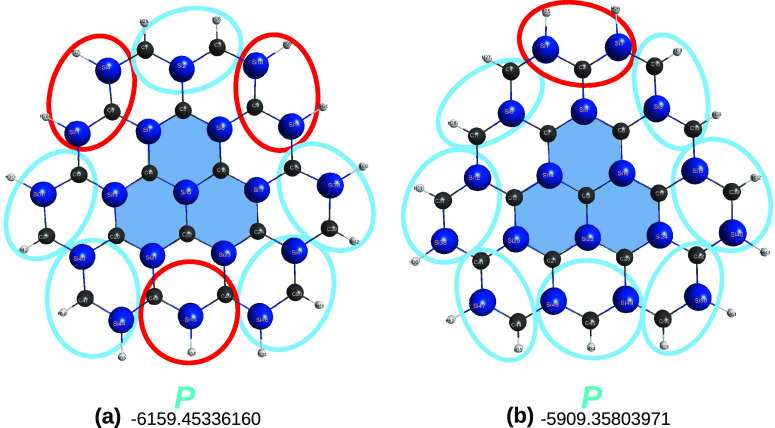
Optimized sila-all-*a*- and sila-all-*b*-[5]triangulenyl structures. Symbols as in Figure 9.

Naturally, one wonders whether the above collected data may also explain the planarity of the sila-*a* and sila-*b*-[5]triangulenyl radicals; Figure 12. The answer is positive, since in both cases we just need to gather the information already used for the corresponding [2-triangulenyl] clusters (now with 2 less H atoms); note that the tiles may always be chosen with the H atom bonded to carbon atoms such as to warrant that they are planar, as noted elsewhere.^13^ One has:
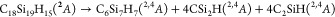
15
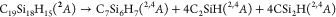
16

**Figure 13:**
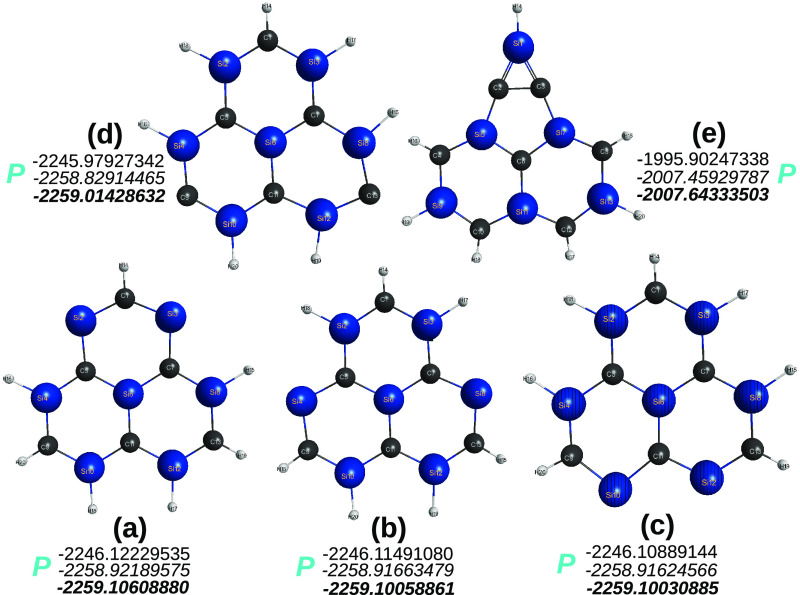
Optimized structures of C_6_Si_7_H_7_ and C_7_Si_6_H_7_ radicals at DFT/M06-2X level of theory with the MINI (top energy), ANO-VT-*D*Z (middle), and ANO-VT-*T*Z (bottom) basis sets. When not specified, the bluish symbols refer to all indicated levels of theory. For the relative energies, see the text.

For the sila-*a* and sila-*b* [5]triangulenyl radicals, we have carried out both B3LYP and M06-2X calculations with the MINI basis sets and found them to be perfectly planar, with no single dihedral angle deviating from 0 or 180°. Similarly, for C_6_Si_7_H_7_(^2,4^*A*), we predicted 4 perfectly planar isomers, of which the one with lowest total energy is reported in panel (a) of Figure 13; for all, the corresponding Cartesian coordinates are given in the SI. The last 3 isomers [panels (b), (c) and (d)] have the 2 missing H atoms in the second and first rows (*a*) of Si atoms from below and in the first row (*b*) of C atoms from below. At the B3LYP level of theory, they lie, in the same order, 4.6, 8.4, and 89.8 kcal mol^–1^ above the one shown in panel (a) of Figure 13. At the M06-2X/ANO-VT-*D*Z level of theory, these energies are in the same order 3.3, 3.6, and 58.2 kcal mol^–1^. Optimizations for the sila-*a* and sila-*b* compounds have further been estimated at the rather expensive M06-2X/ANO-VT-*T*Z level of theory as indicated by the italicized bold numbers in Figure 13, 3.4, 3.6, and 57.6 kcal mol^–1^, in the same order. Except for slightly reversing the relative positioning of the second and third isomers in total energy, no other significantly distinct findings are observed. This is in perfect agreement with what might be predicted from the planarity encountered for the C_18_Si_19_H_15_(^**2**^*A*) and C_19_Si_18_H_15_(^**2**^*A*) parent radicals at the same levels of theory, and with experimental data. Indeed, by enhancing the level of theory to B3LYP and M06-2X with the ANO-VT-*D*Z basis sets yields only slight nonplanarities for the hybrid C/Si clusters C_6_Si_10_ and C_10_Si_6_, as illustrated in Figure 11.

**Figure 14:**
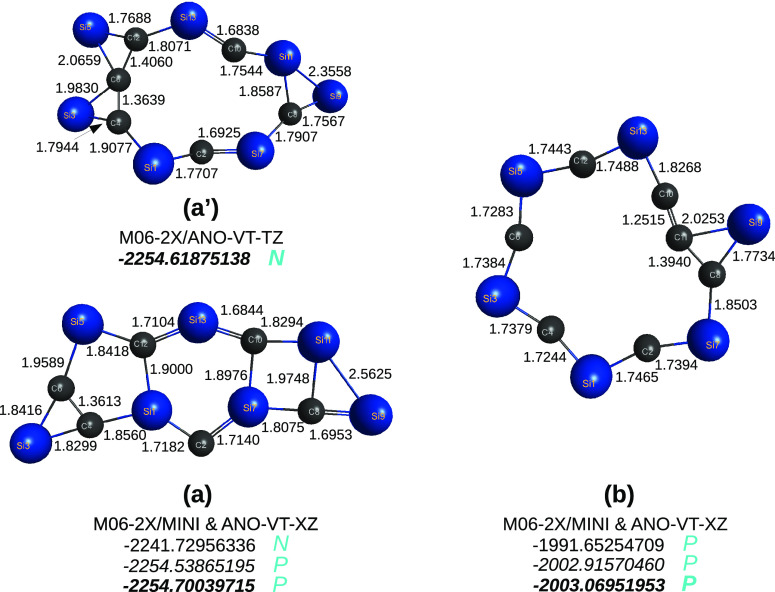
Optimized structures of C_6_Si_7_ and C_7_Si_6_ radicals at DFT/M06-2X using the MINI [top row, panel (a)], ANO-VT-*D*Z [middle row, panel (a)], and ANO-VT-*T*Z [bottom row, panel (a)] levels of theory. Panel (a) refers, therefore, to one of the optimized MINI and one of the ANO-VT-*T*Z structures (the one lowest in energy, with the given parameters); the other, with the ANO-VT-*T*Z basis set, is shown in panel (a′). All three optimizations for the cluster in panel (b) led to basically the same structure (the given parameters are for the ANO-VT-*T*Z basis set).

Alternatively, one could think of using the inner C/Si-clusters, the tiles and molecular hydrogen. Calculations have been performed for such clusters with the M06-2X density functional, the MINI and ANO-VT-*X*Z (X = *D*, *T*) basis sets, with the latter, fairly expensive, converged tightly, typically to better than 10^–5^*E_h_* Å^–1^; see Figure 14. Although the results show some of the clusters tending to assume a planar form, the number of involved H atoms is odd, and hence, the approach is not recommended. It should additionally be noted that the optimized M06-2X/ANO-VT-*T*Z structure of C_6_Si_7_ is nonplanar or quasi-planar (the few typical dihedral deviations from planarity do not exceed 5°) when viewed by the naked eye, depending on somewhat distinct guess structures employed for the optimization. Clearly, although one may conceive to set up one-center expansions that are complete and orthonormal, one should recall that convergence with common basis sets is invariably slow since little additional physics has been built into them. As a result, enhancing the basis set size can hardly warrant the avoidance of introducing unexpected (nonphysical) features. So, we may tentatively attribute such nonplanarity deviations to numerical errors that reflect some unknown unbalanced overlap between the atomic basis sets, hence subbtle attractions or repulsions due to lack of completeness or even the presence of linear dependencies. Not unexpectedly, these results also corroborate the fact that an optimized structure for any but small-size molecules (which afford to be studied with unquestionable accuracy) is always dictated by the method and basis sets that are employed, not to mention the starting guess (recalling that convergence is commonly done to the nearest nearby stationary point).

This said, one may pursue the splitting the sila-*a*-[5]triangulenyl radicals as indicated next for the species in panel (a) of Figure 13,
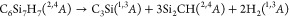
17and corresponding partitions for the other isomers. Analogously, one could write for the sila-*b*-[5]triangulenyl radical in Figure 13(e)
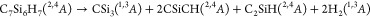
18which shows that both predict the parent molecules to be planar.

A final remark to note that no attempt has been made to consider fully sila-[*n*]triangulenes: all C atoms replaced by Si ones. However, it is foreseable from the results here reported that [*n*]triangulenes, where both *a*- and *b*-type C atoms are substituted by Si atoms, are nonplanar and very much in accord with what is predicted for benzene^12^ and when just a few atoms are replaced.

### 3.4 Other Sila-[*n*]Triangulenes: A Brief Detour

**Figure 15:**
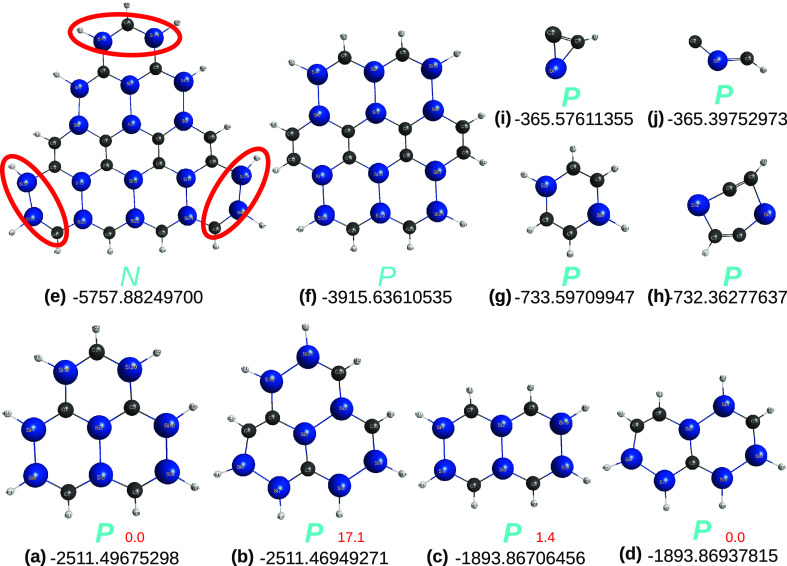
Other optimized sila-molecules studied in the present work: (a, b) isomers of the C_5_Si_8_H_9_ radical; (c, d) isomers of sila-naphthalene C_4_Si_6_H_8_, conjectured to be embedded in the radicals (a) and (b), respectively; (e) variant of sila-[4]triangulene with formula C_15_Si_18_H_15_; (f) variant of sila-coronene, C_12_Si_12_H_12_; (g) triplet state of sila-benzene C_4_Si_2_H_6_; (h) triplet state of sila-benzdiyne C_4_Si_2_H_2_; (i) doublet state of C_2_SiH; (j) quartet state of C_2_SiH. Structures in (a)–(f) were optimized at the DFT/M06-2X/V*D*Z level, while panel (g) employed DFT/M06-2X/AV*T*Z and that in (h) employed MP2/V(*T* + *d*)Z (a similar structure is obtained with the former). In turn, (i) and (j) employed CCSD(T)/AV(*T* + *d*)Z. Also indicated are the corresponding optimized energies, relative energies (in red) in panels (a, b) and (c, d), and *P* vs *N* classification.

It is timely to ask how the approach performs with other, less common, sila-[*n*]triangulenes. An attempt to assess this issue is reported next. As examples, two isomers of the sila-[2]triangulene are first considered. The point to address is how planarity is affected by substituting simultaneously carbon atoms of rows *a* and *b* of the triangulene. The two isomeric structures here considered are illustrated in panels (a) and (b) of Figure 15. First, tightly converged (≤10^–5^*E_h_* Å^–1^) calculations were carried out at the DFT/M06-2X/MINI level. For the isomer in panel (a) of Figure 15, they yielded a planar structure. Conversely, similar quality results for the radical in panel (b) yielded a nonplanar structure, with deviations from planarity in various tetrads of atoms, some attaining 35°. Such trends changed considerably when the basis sets were upgraded to V*D*Z quality, keeping an equally tight convergence. Both structures in panels (a) and (b) of Figure 15 turn now to be essentially planar. Given the exploratory nature of the calculations in this section and the fact that they are largely corroborated from the tiles and generalized quasi-molecules, no further basis set enhancement was deemed justified due to its high computational cost. Instead, we pay attention to panels (c) and (d) of Figure 15, which show the structures of the sila-naphthalene quasi-molecules embedded in the sila-[2]triangulenes (a) and (b), respectively. Both are predicted close to perfect planarity [only one dihedral angle attains 1.9°: (2,5,8,9)]; with MINI, the sila-naphthalene in panel (d) becomes nonplanar (in harmony with the parent sila-[2]triangulene), which reinforces the need for tiles and generalized tiles calculated at an as high as possible level of theory.

Panel (e) of Figure 15 illustrates the sila-[4]triangulene structure here considered, maintaining a tight convergence in the optimization procedure. As in the previous case, it shows C/Si substitutions in both *a* and *b* rows of the triangulene. At the DFT/B3LYP/MINI level, the optimized structure corresponds to a minimum, although 3 out of its 138 real frequencies are low vibrations. It also shows numerous dihedral angles deviating moderately from planarity, while a couple attain 30°. So, the structure is here catalogued as nonplanar (with a light italic *N*; see later). To confirm it, we have performed V*D*Z quality calculations for the sila-coronene structure in panel (f) of Figure 15. This evinces an essentially planar pattern, although 13 tetrads of atoms show small deviations from planarity of up to 2.8°. Due to the high cost of the latter, no attempt was done to calculate the harmonic vibrational frequencies. As a further test, the calculations were redone with tight convergence at the ANO-VT-*D*Z basis set level. No significant differences were observed: e.g., the sila-coronene structure in panel (f) of Figure 15 reveals 11 dihedral deviations still up to 2.8°, while the extremely expensive calculations for the sila-[4]triangulene show only 3 dihedral angles deviating from planarity [2 smaller than 1.5° and the third, for the tetrad (12,36,37,38), reaching ∼8.5°].
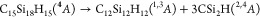
19
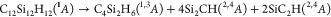
20which shows that the tiles database may not require more than ground-state species to satisfy the spin-spatial correlation rules. However, we can add that C_4_Si_2_H_6_(^3^*A*) remains planar in the triplet state calculated at the M06-2X/AV*T*Z level of theory [see panel (g) of Figure 15] and likewise for its singlet ground-state.^12^

A parenthetical point to note that MP2/MINI calculations for C_4_Si_2_H_6_(^3^*A*) predict a saddle point structure with imaginary frequencies in out-of-the-plane modes, while convergence was reached to a saddle point of index one with the V(*T* + *d*)Z basis set. This recalls the known saga of benzene at the MP2/AV*T*Z level.^12,81,82^ Accordingly, one may attribute the result to lack of static correlation in the MP2 method (which would contrast with DFT, supposed to account for some at the unrestricted level) and to linear dependencies in the basis sets. Since pruning of the basis is not possible with Molpro and the use of ANO-VT-V*X*Z basis sets do not appear to help, we report the M06-2X/AV*T*Z results that predict all frequencies to be real. It should be added that similar planar structures are obtained at the DFT/B3LYP/AV*T*Z level.

Additionally, one expects sila-benzdiyne (C_4_Si_2_H_2_) to be formed in its triplet state, since any triplet or higher spin states formed from the tiles cannot combine with the singlet state to form C_4_Si_2_H_6_(^**3**^*A*). Thence,
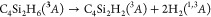
21
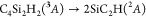
22with eq 22 showing that sila-benzdiyne C_4_Si_2_H_2_(^**3**^*A*) breaks up into two planar-doublet tiles [panel (i) of Figure 15]; it turns out that the quartet tile is also planar [panel (j) of Figure 15]. In summary, the above explains why the sila-molecules in panels (e) and (f) of Figure 15 are expected to be nonplanar and planar, respectively.

### 3.5 Other Spin States: Exploratory Work

We examine now how the spin state of the parent molecule can influence planarity of the sila-[5]triangulenes. Specifically, for its lowest singlet state (see Table 1), the split is as follows:
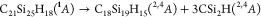
23

If the optimum spin state were the singlet and C_18_Si_19_H_15_ was a doublet, the only possible state for the tetratomic tiles would be the doublet. Since this is not the case, both the doublet and quartet states of the tiles may be involved. In any event, calculations have been performed for the singlet sila-all-*a*- and all-*b*-[5]triangulenes, with planar structures (the only issue at stake) predicted for both.

Of course, one can specify the spin state of the parent molecule, but not for the quasi-molecules. Yet, since the doublet-state of C_18_Si_19_H_15_ would not help in the case of acting the quartet tetratomic tiles, we have performed M06-2X/MINI calculations for its quartet state. A seemingly perfect planar structure is predicted when default convergence is used. It turns out to be a planar stationary point, but a saddle point (recall section 2.2) with a vibrational frequency of 453i cm^–1^ for in-plane distortion. Indeed, a somewhat nonplanar minimum is obtained (with an energy 15.8 kcal mol^–1^ lower than the saddle point) when convergence is further tightned to 5.0 × 10^–6^*E_h_*Å^–2^.

**Figure 16:**
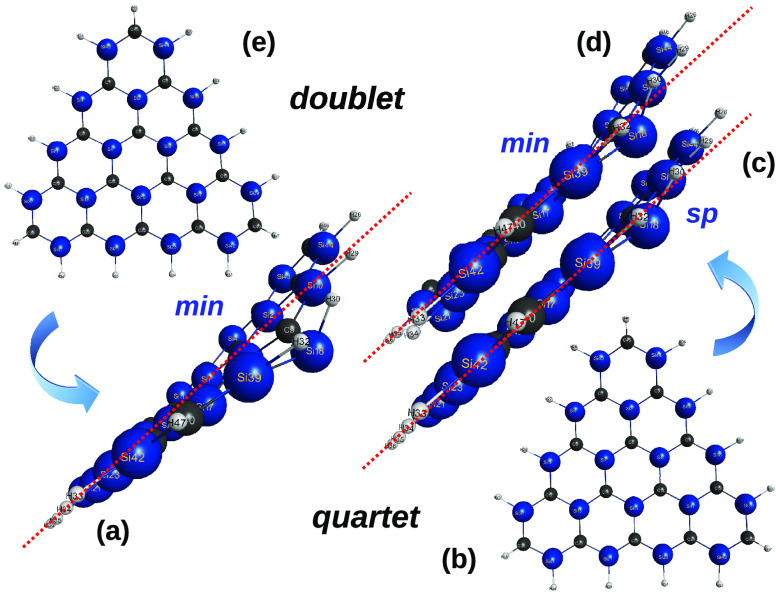
How does the spin-state influence planarity in sila-all-*a*-[4]triangulenes? To guide the eye, red-colored, parallel, straight-line segments are overlapped to the triangulene side view: (a) and (e), doublet state; (b), (c), and (d), quartet state (sp = saddle point, min = minimum). See the text.

A similar observation is valid for the sila-[4]triangulenes,
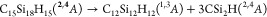
24Figure 16 shows the results of tightly converged calculations for the latter, which show nonplanarity, irrespective of the spin state of the parent molecule. Clearly, differences that are nearly undetectable when overlapping the plots become neat when comparing their side views with a straight-line segment. This also lets one wonder how the above impacts the split in eq 24: will nonplanarity manifest in the C_12_Si_12_H_12_ generalized tile? It turns out that both structures are minima, irrespective of their spin state. The singlet state species is perfectly planar, with a total energy of *E* = −3915.79640309 *E_h_* at the M06-2X/MINI level of theory. Conversely, at the same level of theory, the optimized triplet structure is nonplanar (*E* = −3915.72166782 *E_h_*, thus lying 46.1 kcal mol^–1^ above the singlet), with various dihedrals well away from planarity. Since both contribute to the parent molecule, this may explain why the latter is not planar.

## 4 Concluding Remarks

In a recent paper,^12^ we argued that the planar shape of a molecule can be deciphered from the quasi-tetratomics (tiles) embedded in it. Because the latter can afford to be studied at the highest levels of accuracy currently at our disposal, a tiles database can offer a secure route for examining planarity. Before such a database is completed and the analysis efficiently workable in an automated way under computer supervisioning, an alternative is to employ the concept of a generalized quasi-molecule.^13^ This involves splitting any large molecule into generalized quasi-molecules in which smaller (hence, more amenable to be studied with accurate methods) can subsequently be used to decipher the planarity of the parent molecule. We have here employed this strategy to discuss the shape of [*n* ≤ 5]triangulenes and their sila-derivatives, a question that can hardly find a direct answer via accurate ab initio methods. Although the results largely support the predictions from DFT calculations, they offer a fresh view while promising a consistent route to even larger [*n*]triangulenes and derivatives. Because other spin states of the parent molecule commonly correlate with the same tiles, the prediction made when considering the optimum (highest) spin state is expected to remain largely valid: no structural changes are expected to depend on the parent spin state. Unfortunately, to our knowledge, no experimental information is available on this matter. By the same token, one wonders whether the present approach can help in explaining how a planar molecule changes^86^ after induction of a void by removing one or more atoms. This and other topics (e.g., how to extend to the boron–nitrogen–hydrogen chemistry, also popular in materials science) are assets for future work. Indeed, even less is known about such molecules, and the availability of a likelihood index of planarity could help with assessing synthesization prior to high level calculations becoming affordable.

## Supporting Information Available

Cartesian coordinates and harmonic vibrational frequencies at DFT level with the B3LYP and M06-2X functionals for the optimized structures in Figures 3 and 4; Cartesian coordinates of optimized geometries at RCCSD(T)/cc-pV*T*Z and RCCSD(T)/cc-pV*T*Z levels of theory for the structures in Figure 5; Cartesian coordinates and harmonic vibrational frequencies at RCCSD(T)/V*D*Z and DFT/M06-2X/AV*T*Z levels of theory for the optimized structures in Figure 6; Cartesian coordinates and harmonic vibrational frequencies at DFT/M06-2X/AV*T*Z level for the optimized structures in Figure 7; Cartesian coordinates and harmonic vibrational frequencies at DFT/M06-2X/ANO-VT-TZ level for the optimized structures in Figure 13 (PDF)
