# Supplementary material for: Carbon-[n]Triangulenes and Sila-[n]Triangulenes: Which Are Planar?
Source: J Phys Chem A. 2023 May 31;127(23):5048–64. doi: 10.1021/acs.jpca.3c01820 (PMC10278145; doi:10.1021/acs.jpca.3c01820)
Supplement: Supplementary file 1 — jp3c01820_si_001.pdf [file jp3c01820_si_001.pdf]

Supporting Information: Carbon-[n]Triangulenes and Sila-[n]Triangulenes: Which Are Planar?

A. J. C. Varandas*,†,‡,¶

†*School of Physics and Physical Engineering, Qufu Normal University, 273165, P.R. China*
‡*Department of Physics, Universidade Federal do Espírito Santo, 29075-910 Vitória, Brazil*
¶*Department of Chemistry and Coimbra Chemistry Centre, University of Coimbra 3004-535 Coimbra, Portugal.*

E-mail: varandas@uc.pt

Figure 3:

Panel (a), B3LYP:

22 UKS-SCF000/VDZ ENERGY=-751.79086730 C -0.4422763853 5.1309840874 -0.0764884789 C -1.7355594999 4.5450402386 0.0264954946 C 0.8456964205 4.5298487950 -0.1569303218 C -1.9631035446 3.1352609483 0.0693157034 C 1.0604726356 3.1175145702 -0.1458568632 Si -0.4554095821 2.2518087418 -0.0214550216 C -3.0754019791 2.2438526588 0.1654460891 C -2.9489389182 0.8260293569 0.1835278006 C -1.7943378555 -0.0040164429 0.1168626455 C -0.4625753132 0.5021412797 0.0119573240 C 0.8646870965 -0.0193291218 -0.0732568237 C 2.1651422298 2.2136341419 -0.2078456362 C 2.0265377736 0.7973379273 -0.1715420369 H -0.4373213287 6.2247908059 -0.0973040593 H -2.5807216016 5.2433989287 0.0734274979 H 1.6971363286 5.2182259349 -0.2304239927 H -4.0970366582 2.6390082527 0.2305325455 H -3.8963717376 0.2846082548 0.2616525861 H -1.9735572542 -1.0861043513 0.1503907151 H 1.0343078020 -1.1034516219 -0.0652549668 H 2.9691844740 0.2450379297 -0.2286894298 H 3.1900167677 2.5974261051 -0.2881432809

Low Vibration Wavenumber Nr [1/cm] 1 0.00 2 0.00 3 0.00 4 0.00 5 0.00 6 0.00 7 37.75

Vibration Wavenumber Nr [1/cm] 1 181.84 2 182.08 3 300.71 4 343.25 5 343.70 6 362.95 7 397.87 8 397.93 9 424.09 10 424.29 11 477.32 12 567.19 13 578.60 14 578.74 15 593.63 16 739.59 17 744.28 18 744.52 19 774.76 20 774.91 21 795.93 22 864.12 23 864.24 24 881.79 25 909.62 26 909.81 27 967.00 28 967.15 29 976.21 30 997.45 31 997.60 32 1028.71 33 1150.63 34 1176.18 35 1176.49 36 1230.41 37 1231.63 38 1231.74 39 1266.87 40 1267.51 41 1302.36 42 1305.64 43 1396.60 44 1397.28 45 1455.00 46 1455.08 47 1469.71 48 1476.29 49 1518.65 50 1518.77 51 3119.81 52 3120.13 53 3121.69 54 3123.14 55 3123.71 56 3123.82 57 3167.75 58 3167.93 59 3168.76

Panel (a), B3LYP:

22 UKS-SCF000/VDZ ENERGY=-1003.11401556 C -0.4423439506 5.1229685350 -0.0764158743 C -1.7339580176 4.5361085555 0.0265969060 C 0.8440267824 4.5212417566 -0.1568480531 C -1.9662939861 3.1271840338 0.0695470909 C 1.0638959356 3.1097179325 -0.1463175182 Si -0.4551841636 2.2197251430 -0.0216653950 C -3.1337163665 2.3133987929 0.1684162077 C -3.2029764696 0.8852369004 0.2004520224 C -2.2743501697 -0.1923280502 0.1544778436 Si -0.4643943234 0.1090124566 0.0191812166 C 1.3428351640 -0.2131838380 -0.1036388814 C 2.2241384185 2.2825283956 -0.2133320177 C 2.2808767426 0.8536509885 -0.1905741262 H -0.4375284529 6.2169193495 -0.0971360305 H -2.5794935811 5.2349681497 0.0737777432 H 1.6957407958 5.2102711832 -0.2302681420 H -4.1108021480 2.8102090819 0.2288544300 H -4.2387659161 0.5316170589 0.2812473306 H -2.7264396694 -1.1882054133 0.2057137534 H 1.7861913837 -1.2142121037 -0.1163327151 H 3.3135398539 0.4881505852 -0.2572563713 H 3.2055720081 2.7680679264 -0.2920619297

Imaginary Vibration Wavenumber Nr [1/cm] 1 68.77

Vibration Wavenumber Nr [1/cm] 1 121.24 2 164.82 3 187.50 4 277.57 5 280.33 6 282.33 7 345.55 8 358.29 9 358.88 10 417.71 11 434.16 12 515.76 13 519.11 14 563.98 15 574.01 16 637.61 17 682.94 18 687.44 19 697.71 20 701.85 21 765.80 22 781.27 23 808.70 24 815.48 25 837.39 26 855.53 27 863.54 28 911.67 29 964.22 30 968.23 31 978.50 32 986.02 33 1002.39 34 1025.66 35 1158.13 36 1180.80 37 1216.25 38 1224.21 39 1249.29 40 1257.93 41 1271.96 42 1280.52 43 1311.31 44 1366.01 45 1416.52 46 1430.77 47 1453.03 48 1453.65 49 1492.36 50 1507.50 51 3107.40 52 3107.57 53 3113.81 54 3116.47 55 3127.38 56 3127.73 57 3154.82 58 3156.08 59 3167.00

Panel (c), B3LYP:

22 UKS-SCF000/VDZ ENERGY=-1254.46329385 C -0.0539084480 6.1273173369 -0.0222193143 C -1.3633183039 5.5819501701 -0.0325292448 C 1.2441953638 5.5868221394 -0.2090788728 Si -1.6816711935 3.8342337332 -0.3728487240 Si 1.5205785243 3.8406637337 -0.5910591198 Si -0.1491203129 2.7321335315 -1.5204667458 C -2.8930903176 2.5973930988 0.2186485122 C -2.5203196108 1.2423021348 0.2475754839 C -1.2817903180 0.5860806460 -0.0199929379 C -0.0759819553 1.1644533717 -0.5015603177 C 1.1872793352 0.5905941944 -0.1913637657 C 2.8064764233 2.6093745375 -0.1705139737 C 2.4474281438 1.2523672856 -0.0936309614 H -0.0355950224 7.1863201170 0.2765836960 H -2.1517262892 6.2570567940 0.3151184828 H 2.0697813209 6.2647901164 0.0296319731 H -3.8679117884 2.8351886044 0.6579220477 H -3.2813403715 0.5656635519 0.6582487428 H -1.2318835366 -0.4516677489 0.3385667397 H 1.1920435585 -0.4481741655 0.1675083928 H 3.2602431691 0.5788628448 0.2086004394 H 3.8305835092 2.8515540523 0.1332769378

Vibration Wavenumber Nr [1/cm] 1 114.62 2 115.31 3 123.17 4 206.07 5 208.70 6 223.27 7 242.73 8 256.81 9 296.35 10 334.36 11 372.89 12 399.42 13 437.41 14 466.40 15 477.61 16 530.14 17 545.59 18 586.80 19 598.43 20 646.50 21 658.55 22 676.24 23 699.00 24 708.26 25 722.87 26 780.38 27 794.75 28 795.52 29 815.43 30 822.62 31 868.03 32 986.20 33 987.02 34 993.00 35 1004.52 36 1036.81 37 1048.15 38 1175.63 39 1196.88 40 1202.83 41 1212.30 42 1265.09 43 1292.00 44 1295.72 45 1314.11 46 1355.99 47 1409.01 48 1443.41 49 1450.48 50 1466.95 51 1495.50 52 3086.44 53 3095.82 54 3098.01 55 3120.27 56 3121.22 57 3150.91 58 3151.43 59 3152.09

60 3156.12

Panel (d), B3LYP:

22 UKS-SCF000/VDZ ENERGY=-1254.51203389 C -0.4406544090 5.5054844453 -0.0836272611 C -1.7295987763 4.9339375136 0.0189354646 C 0.8431701692 4.9191002189 -0.1642597844 Si -1.9766047921 3.1432046883 0.0701189259 Si 1.0743762836 3.1256126862 -0.1472025851 C -0.4549981274 2.2514579978 -0.0216644736 C -3.4103124532 2.0476633441 0.1931039258 C -3.2735518771 0.6407192036 0.2100266368 C -2.1337551314 -0.1930715568 0.1443372405 Si -0.4627427871 0.4857312694 0.0122135266 C 1.2022278958 -0.2123123839 -0.0934184346 C 2.4983849859 2.0136102520 -0.2279630692 C 2.3492791068 0.6083083047 -0.1908308297 H -0.4358222399 6.6049141312 -0.1044404282 H -2.5651251034 5.6384988496 0.0652716260 H 1.6849323465 5.6139058638 -0.2372691861 H -4.4324934079 2.4317152564 0.2586539617 H -4.2259244083 0.0966360377 0.2885317044 H -2.3236911284 -1.2698595026 0.1780692614 H 1.3828003470 -1.2912150214 -0.0859238606 H 3.2967817591 0.0531686584 -0.2480998553 H 3.5238916180 2.3858371637 -0.3081450157

Vibration Wavenumber Nr [1/cm] 1 79.51 2 125.34 3 125.35 4 224.31 5 255.65 6 255.78 7 274.09 8 274.09 9 310.80 10 310.86 11 315.58 12 363.43 13 446.27 14 456.85 15 467.33 16 467.56 17 591.79 18 611.56 19 611.64 20 676.89 21 677.10 22 690.08 23 754.13 24 754.18 25 772.86 26 773.09 27 784.76 28 798.85 29 799.00 30 814.84 31 869.23 32 994.81 33 995.16 34 996.87 35 1044.77 36 1045.57 37 1059.89 38 1082.45 39 1082.48 40 1187.55 41 1191.04 42 1191.81 43 1272.60 44 1294.84 45 1295.00 46 1378.34 47 1378.62 48 1379.54 49 1436.31 50 1458.32 51 1458.58 52 3094.25 53 3094.35 54 3094.66 55 3164.83 56 3165.52 57 3165.77 58 3168.42 59 3168.74 60 3169.81

Panel (e), B3LYP:

22 UKS-SCF000/VDZ ENERGY=-1505.10208038 C -0.0579435008 6.1446951255 -0.0842154951 C -1.4101027325 5.6603731155 0.0130047515 C 1.3046242425 5.6875540353 -0.1636606894 Si -1.9604885642 3.9348033668 0.0724925112 Si 1.8936607718 3.9739037930 -0.1584893711 Si -0.0214983144 2.8385635350 -0.0361317580 C -3.1455039871 2.5687856652 0.1939745287 C -2.8569624917 1.1584133364 0.2035291656 C -1.7606774735 0.2267509823 0.1477712998 Si 0.0049051081 0.6092290846 0.0065168602 C 1.7805728960 0.2619735918 -0.0955999508 C 3.1101132681 2.6324111559 -0.2263801605 C 2.8548689654 1.2162464239 -0.1835058738 H -0.0703192458 7.2439622295 -0.1042909899 H -2.1515334501 6.4659886932 0.0492375039 H 2.0281832128 6.5076093462 -0.2273379226 H -4.2163949409 2.7866959608 0.2701850500 H -3.8000743985 0.5990864416 0.2848495108 H -2.0850142086 -0.8183336481 0.1992224241 H 2.1294048568 -0.7763416421 -0.0965023065 H 3.8115395437 0.6769271677 -0.2363368643 H 4.1761164228 2.8727329702 -0.3019147538

Imaginary Vibration Wavenumber Nr [1/cm] 1 69.64

Vibration Wavenumber Nr [1/cm] 1 63.05 2 65.39 3 150.71 4 151.83 5 169.43 6 181.60 7 187.59 8 190.46 9 276.05 10 277.67 11 322.42 12 322.59 13 334.15 14 383.95 15 388.27 16 510.55 17 544.06 18 545.48 19 646.38 20 650.05 21 698.52 22 699.05 23 723.78 24 724.20 25 724.85 26 755.65 27 757.51 28 784.46 29 795.76 30 796.16 31 821.32 32 847.91 33 962.10 34 962.51 35 994.05 36 1008.88 37 1013.76 38 1025.71 39 1251.37 40 1259.65 41 1271.06 42 1274.77 43 1275.27 44 1339.30 45 1339.66 46 1343.79 47 1368.87 48 1441.54 49 1445.85 50 1449.51 51 3120.22 52 3120.46 53 3120.56 54 3164.20 55 3164.37 56 3165.34 57 3167.73 58 3168.02 59 3169.03

Panel (c), M06-2X:

22 UKS-SCF000/VDZ ENERGY=-1254.61040887 C -0.0444394945 6.0846316870 -0.0008413260 C -1.3502224783 5.5383338695 -0.0055088301 C 1.2423745564 5.5600321310 -0.2487937930 Si -1.6387460680 3.8189542620 -0.4014439228 Si 1.4779890240 3.8268929229 -0.6388137982 Si -0.1581982723 2.7174332514 -1.6121106690 C -2.8791289306 2.5987949355 0.1749565215 C -2.5155436545 1.2573446926 0.2408295631 C -1.2668121531 0.6109213975 -0.0160838678 C -0.0798070967 1.1887923300 -0.5286747902 C 1.1683020463 0.6216383614 -0.1789027771 C 2.7813166555 2.6208715752 -0.1978011124 C 2.4343466626 1.2781116143 -0.0827124756 H -0.0181911985 7.1205173848 0.3613756267 H -2.1269992462 6.1797065434 0.4169033415 H 2.0755445633 6.2220103205 -0.0056163978 H -3.8613106162 2.8538285862 0.5798764034 H -3.2673488053 0.5859264705 0.6737357870 H -1.1901863388 -0.4046324828 0.3898264651 H 1.1525474164 -0.3938206655 0.2347813918 H 3.2383770542 0.6164152655 0.2623675038 H 3.7970882543 2.8825756271 0.1090686266

Vibration Wavenumber Nr [1/cm] 1 70.22 2 98.47 3 115.07 4 189.56 5 205.32 6 218.78 7 243.59 8 267.52 9 296.35 10 336.64 11 368.29 12 400.87 13 423.44 14 456.80 15 475.75 16 527.64 17 538.13 18 586.34 19 605.66 20 610.27 21 647.26 22 678.99 23 710.00 24 717.97 25 745.87 26 780.13 27 796.31 28 811.24 29 833.59 30 844.30 31 892.05 32 982.37 33 988.84 34 1010.55 35 1044.00 36 1072.91 37 1099.89 38 1159.63 39 1181.71 40 1210.25 41 1230.26 42 1270.97 43 1300.94 44 1308.96 45 1338.25 46 1404.30 47 1408.04 48 1452.16 49 1455.93 50 1504.02 51 1531.24 52 3125.80 53 3133.31 54 3139.35 55 3155.93 56 3158.60 57 3193.90 58 3199.13 59 3203.15 60 3207.03

Panel (d), M06-2X:

22 UKS-SCF000/VDZ ENERGY=-1254.66238163 C -0.4410969008 5.4838433454 -0.0772685410 C -1.7206930404 4.9188905094 0.0913188515 C 0.8332471907 4.9028167045 -0.2292882706 Si -1.9649562164 3.1362986774 0.0977956738 Si 1.0641347824 3.1181384439 -0.1857527583 C -0.4544257939 2.2493225498 -0.0292739939 C -3.3958708060 2.0520276927 0.1939993051 C -3.2631017286 0.6488269226 0.1728101993 C -2.1270097799 -0.1833275154 0.1179695540 Si -0.4617379699 0.4931678144 0.0170426134 C 1.1969643342 -0.2021034814 -0.0492598918 C 2.4837657682 2.0162783759 -0.2500374874 C 2.3392974626 0.6161828074 -0.1650518466 H -0.4349184617 6.5793193494 -0.0926014302 H -2.5431897577 5.6258500883 0.2132908904 H 1.6607424981 5.6014036269 -0.3649740665 H -4.4115026571 2.4455799938 0.2559720361 H -4.2142775203 0.1025989983 0.2076425353 H -2.3246607723 -1.2560920219 0.1269828413 H 1.3814597678 -1.2755987431 0.0101775170 H 3.2863998711 0.0623676222 -0.1936983209 H 3.5019995999 2.3972556596 -0.3413779200

Vibration Wavenumber Nr [1/cm] 1 67.33 2 119.11 3 127.81 4 211.63 5 253.52 6 254.68 7 270.02 8 283.00 9 309.84 10 310.43 11 316.27 12 359.53 13 457.17 14 457.93 15 487.90 16 495.93 17 595.00 18 596.00 19 598.93 20 626.73 21 667.56 22 686.01 23 750.89 24 752.06 25 765.00 26 777.07 27 785.42 28 804.53 29 819.36 30 836.13 31 878.42 32 997.25 33 1049.24 34 1054.24 35 1060.50 36 1070.26 37 1087.09 38 1113.51 39 1114.44 40 1194.29 41 1204.11 42 1206.73 43 1277.64 44 1292.29 45 1294.16 46 1395.87 47 1397.82 48 1403.44 49 1444.69 50 1465.58 51 1469.71 52 3137.53 53 3137.76 54 3150.39 55 3209.82 56 3211.45 57 3212.95 58 3213.61 59 3216.29 60 3216.70

Panel (e), M06-2X:

22 UKS-SCF000/VDZ ENERGY=-1505.33376709 C -0.0604299585 6.0202530033 0.1478471791 C -1.3212319822 5.4248923077 -0.0137527510 C 1.1944222875 5.4297655695 -0.0858198646 Si -1.4953691774 3.6899244437 -0.5949303285 Si 1.3490520675 3.7198531112 -0.7303967231 Si -0.1450652088 2.9118914311 -2.2468434425 C -2.7730126917 2.5817159997 0.1465672224 C -2.5956831917 1.2355500090 0.3988157420 C -1.4374021744 0.4596196356 0.1058972635 Si -0.0628559789 1.0204810648 -0.9184294281 C 1.4413654084 0.4849544610 -0.0844175644 C 2.7588956420 2.6148092779 -0.2642747773 C 2.6345275887 1.2646558386 -0.0084842978 H -0.0507163398 7.0348565484 0.5592725744 H -2.1954670952 6.0115204065 0.2892985698 H 2.0833497299 6.0120493620 0.1828320921 H -3.7352042450 3.0199852520 0.4393890971 H -3.3933286847 0.7208938103 0.9479575301 H -1.3625955917 -0.5122240323 0.6107285936 H 1.4388494794 -0.4644024258 0.4651111134 H 3.5250858165 0.7373031198 0.3558219687 H 3.7602902803 3.0536825359 -0.1757722991

Vibration Wavenumber Nr [1/cm] 1 74.76 2 92.04 3 131.11 4 164.75 5 185.89 6 190.52 7 205.06 8 243.20 9 251.91 10 274.46 11 281.00 12 301.14 13 386.00 14 405.00 15 416.50 16 424.19 17 438.25 18 446.72 19 564.33 20 593.44 21 632.39 22 676.05 23 699.70 24 718.69 25 723.54 26 743.34 27 747.39 28 774.69 29 792.20 30 825.01 31 827.57 32 841.14 33 860.59 34 1006.02 35 1030.03 36 1059.43 37 1070.90 38 1092.44 39 1101.30 40 1171.84 41 1194.11 42 1224.54 43 1246.20 44 1318.02 45 1350.44 46 1383.64 47 1395.35 48 1397.19 49 1414.09 50 1510.97 51 1545.49 52 3162.70 53 3163.80

54 3173.34 55 3174.12 56 3175.18 57 3181.63 58 3182.50 59 3184.74 60 3192.67

Figure 4

Panel (a), B3LYP:

18 UKS-SCF000/VDZ ENERGY=-636.98382886 C -2.1228748405 3.1858993629 0.0902556495 C 1.2492960284 3.1545924399 -0.1563559877 Si -0.4416427720 2.5665351159 -0.0228233578 C -3.1085043943 2.2099274133 0.1795338580 C -2.9288916813 0.7937747321 0.1882547024 C -1.7184373414 0.1199639375 0.1099194950 C -0.4561596645 0.7666340239 0.0065838948 C 0.7954890049 0.0966127358 -0.0748828869 C 2.2191216757 2.1604576305 -0.2121361733 C 2.0166845395 0.7478506236 -0.1747645997 H -2.4204933780 4.2372886614 0.0979746142 H 1.5639624823 4.2001660655 -0.1968684713 H -4.1488478072 2.5507951013 0.2517828007 H -3.8381432553 0.1918005579 0.2661892017 H -1.7419106587 -0.9776450361 0.1296849103 H 0.8011973118 -1.0012915832 -0.0583936126 H 2.9161527260 0.1290324664 -0.2317109158 H 3.2648522745 2.4820871815 -0.2943543615

Vibration Wavenumber Nr [1/cm] 1 133.39 2 169.01 3 278.28 4 306.10 5 361.46 6 384.40 7 415.48 8 440.72 9 513.85 10 569.36 11 586.19 12 641.55 13 708.89 14 724.48 15 751.61 16 833.62 17 837.77 18 847.30 19 874.18 20 890.28 21 962.30 22 982.19 23 984.89 24 986.58 25 1004.59 26 1008.08 27 1158.54 28 1160.12 29 1192.90 30 1212.31 31 1244.57 32 1310.81 33 1332.42 34 1358.61 35 1424.61 36 1444.25 37 1510.40 38 1544.98 39 1597.45 40 1597.59 41 3115.75 42 3117.22 43 3125.50 44 3125.76 45 3174.55 46 3174.85 47 3182.32 48 3183.56

Panel (b), B3LYP:

18 UKS-SCF000/VDZ ENERGY=-888.30550352 C -2.1503602159 3.2098404881 0.0918599034 C 1.2766825190 3.1784481650 -0.1588074558 Si -0.4415935617 2.6161428654 -0.0262969974 C -3.1807686544 2.2792747567 0.1832957801 C -3.1922230499 0.8391501208 0.2068752507 C -2.1767112366 -0.1102052692 0.1470193725 Si -0.4587090496 0.4518705920 0.0096617574 C 1.2504372481 -0.1415736759 -0.1044074561 C 2.2923170881 2.2291867868 -0.2180935751 C 2.2809336282 0.7890828725 -0.1942518446 H -2.4423774539 4.2650877799 0.0979296141 H 1.5854101463 4.2281952811 -0.1984276088 H -4.1888610557 2.7068608514 0.2521713575 H -4.2069361425 0.4301426086 0.2900675582 H -2.4853978613 -1.1599684720 0.1874697768 H 1.5426115979 -1.1968180109 -0.1087476067 H 3.2891711888 0.3614994173 -0.2610195369 H 3.3072251150 2.6382642726 -0.2984095293

Vibration Wavenumber Nr [1/cm] 1 96.96 2 153.21 3 182.57 4 217.25 5 283.21 6 296.68 7 334.72 8 373.50 9 425.47 10 497.28 11 529.67 12 567.05 13 637.30 14 649.51 15 666.23 16 768.84 17 787.71 18 799.97 19 809.37 20 818.92 21 844.92 22 860.13 23 949.36 24 949.59 25 950.35 26 958.03 27 1005.18 28 1005.22 29 1180.29 30 1181.44 31 1228.11 32 1234.99 33 1336.10 34 1353.08 35 1363.10 36 1384.53 37 1455.36 38 1510.64 39 1534.47 40 1543.59 41 3110.97 42 3111.33 43 3130.71 44 3130.89 45 3155.24 46 3156.27 47 3158.02 48 3159.04

Panel (c), B3LYP:

18 UKS-SCF000/VDZ ENERGY=-1139.66762229 Si -2.3284164781 3.5081268835 0.1028915709 C 1.5575121285 3.1677837317 -0.1793348947 Si -0.2265183576 2.8164019620 -0.0438776787 C -3.4621053563 2.1273648540 0.2101009734 C -3.2754621287 0.7207550225 0.2165492184 C -2.1212297986 -0.0647687749 0.1409942707 Si -0.4676473205 0.6347428186 0.0062358263 C 1.1972864832 -0.0956256744 -0.1051770426 C 2.4808707132 2.1388840433 -0.2317025844 C 2.3230899976 0.7047469526 -0.1992832125 H -2.9151360303 4.8777173660 0.1280911485 H 1.9503125122 4.1887865855 -0.2230658222 H -4.5115871377 2.4418417074 0.2840754612 H -4.2117026379 0.1508151542 0.2975445281 H -2.2684876064 -1.1494900626 0.1696612383 H 1.3695744716 -1.1779990041 -0.1019595775 H 3.2809432140 0.1732844923 -0.2613603208 H 3.5295535820 2.4511133723 -0.3124943422

Vibration Wavenumber Nr [1/cm] 1 61.98 2 116.92 3 156.36 4 184.88 5 230.02 6 252.35 7 311.10 8 312.65 9 383.75 10 390.43 11 464.91 12 479.10 13 533.42 14 537.11 15 636.74 16 655.65 17 673.05 18 702.66 19 729.49 20 763.45 21 787.29 22 788.84 23 812.55 24 834.37 25 846.12 26 955.71 27 963.83 28 989.09 29 1010.36 30 1081.82 31 1184.67 32 1204.30 33 1228.13 34 1325.32 35 1359.85 36 1382.06 37 1397.42 38 1468.31 39 1514.65 40 1560.39 41 2223.24 42 3096.31 43 3111.94 44 3125.58 45 3129.97 46 3147.99 47 3154.28 48 3159.77

Panel (d), B3LYP:

18 UKS-SCF000/VDZ ENERGY=-1139.67491884 Si -2.5470707294 3.4548486605 0.1188695799 Si 1.6773961069 3.4162627629 -0.1923081280 Si -0.4400523067 2.7971924807 -0.0283145521 C -3.5203549625 1.9686601150 0.2135784060 C -2.9949676587 0.6550674168 0.1954875948 C -1.6759897499 0.2119135282 0.1053226920 C -0.4546382011 0.9452293573 0.0037349265 C 0.7549149218 0.1896696633 -0.0726162569 C 2.6268952682 1.9124826366 -0.2378843970 C 2.0808330561 0.6086456304 -0.1764311656 H -3.1517977924 4.8112002354 0.1409128617 H 2.3035220041 4.7612404785 -0.2623210434 H -4.6107859865 2.0470577325 0.2927979524 H -3.7370819714 -0.1492170547 0.2627056806 H -1.5563774482 -0.8790512032 0.1139701053 H 0.6176856185 -0.8988863068 -0.0447096872 H 2.8102645166 -0.2089709615 -0.2162146361 H 3.7184555248 1.9711357381 -0.3186911429

Low Vibration Wavenumber Nr [1/cm] 1 0.00 2 0.00 3 0.00 4 0.00 5 0.00 6 0.00 7 40.77

Vibration Wavenumber Nr [1/cm] 1 106.53 2 132.66 3 207.93 4 247.33 5 263.21 6 357.41 7 359.62 8 369.88 9 393.37 10 396.59 11 422.95 12 556.77 13 593.77 14 600.18 15 601.79 16 670.59 17 689.16 18 712.31 19 714.86 20 745.41 21 763.12 22 833.30 23 851.05 24 855.59 25 897.31 26 988.75 27 998.65 28 1027.47 29 1073.45 30 1182.78 31 1218.75 32 1249.11 33 1316.52 34 1326.28 35 1361.88 36 1474.23 37 1486.66 38 1505.87 39 1571.60 40 2249.96 41 2252.05 42 3110.20 43 3113.86 44 3132.22 45 3133.50 46 3150.35 47 3150.74

Panel (e), B3LYP

18 UKS-SCF000/VDZ ENERGY=-1139.67764952 Si -2.0868091932 3.2681990825 0.0863730475 Si 1.2141043094 3.2380248655 -0.1554575885 C -0.4417583308 2.5718499895 -0.0233538097 C -3.4288946466 2.1237099659 0.2039156830 C -3.2557951109 0.7115554487 0.2140106162 C -2.0745610698 -0.0219720941 0.1386950504 Si -0.4560893244 0.7597239838 0.0069385025 C 1.1497882977 -0.0514917327 -0.0978701563 C 2.5378869385 2.0691177292 -0.2338598612 C 2.3424906692 0.6603221609 -0.1968938708 H -2.3333746599 4.7360915954 0.0806772490 H 1.4837596731 4.7012748109 -0.1984350134 H -4.4545616138 2.4978229665 0.2734453315 H -4.1825255090 0.1253240278 0.2920053910 H -2.1546111899 -1.1129161554 0.1622163716 H 1.2126100524 -1.1437377806 -0.0847794897 H 3.2598352724 0.0571927582 -0.2545695009 H 3.5693558756 2.4243917679 -0.3151691622

Vibration Wavenumber Nr [1/cm] 1 105.79 2 114.83 3 199.11 4 245.63 5 265.67 6 291.74 7 317.64 8 335.73 9 369.34 10 450.00 11 451.83 12 466.82 13 473.12 14 482.18 15 603.38 16 676.84 17 707.09 18 712.74 19 717.91 20 726.60 21 772.33 22 775.77 23 790.49 24 806.25 25 854.83 26 897.17 27 951.43 28 1000.19 29 1003.29 30 1062.17 31 1070.13 32 1073.31 33 1172.28 34 1176.08 35 1307.77 36 1336.13 37 1398.75 38 1401.71 39 1487.81 40 1533.25 41 2236.27 42 2241.48 43 3099.01 44 3099.07 45 3163.62 46 3165.00 47 3168.84 48 3169.18

Panel (f), B3LYP:

18 UKS-SCF000/VDZ ENERGY=-1391.02994695 Si -2.5682968513 3.4784945714 0.1845572634 Si 1.7118380898 3.4348767292 -0.1355022623 Si -0.4695659523 3.0324077211 -0.4711862377 C -3.6102013801 2.0351983389 0.2575396091 C -3.2865784088 0.6493701154 0.2451405032 C -2.0655447481 -0.0021072310 0.0940310166 Si -0.4664846001 0.8171589303 -0.1304312107 C 1.1312402426 -0.0346510133 -0.1452568694 C 2.7239014920 1.9708684698 -0.2133881279 C 2.3740830401 0.5918196816 -0.1777070256 H -3.2582104117 4.8007659060 0.1626622638 H 2.4174391688 4.7431344118 -0.2591608229 H -4.6743723873 2.2545750527 0.4128958550 H -4.1524191091 -0.0126606364 0.3873794572 H -2.0919342322 -1.0962810241 0.1567905179 H 1.1445768761 -1.1292543447 -0.0859265725 H 3.2378956119 -0.0878343517 -0.1649209578 H 3.8034838096 2.1686001029 -0.2196276393

Vibration Wavenumber Nr [1/cm] 1 56.86 2 102.09 3 143.78 4 157.76 5 211.19 6 228.86 7 234.32 8 295.21 9 326.52 10 359.00

11 406.42 12 408.76 13 468.68 14 475.10 15 475.29 16 571.56 17 615.44 18 668.17 19 681.20 20 688.86 21 700.04 22 710.42 23 774.19 24 776.21 25 790.57 26 793.62 27 816.23 28 854.14 29 997.39 30 999.42 31 1079.03 32 1084.45 33 1198.64 34 1203.62 35 1316.50 36 1349.41 37 1400.15 38 1400.54 39 1480.78 40 1528.13 41 2214.97 42 2217.69 43 3095.91 44 3096.19 45 3130.17 46 3130.38 47 3141.99 48 3143.12

Figure 5

6 RCCSD(T)/CC-PV(T+D)Z,H=CC-PVTZ ENERGY=-404.08362130 C 2.2473213418 1.2347527606 0.5737515202 C 1.3119620860 -1.3494232307 -0.9571860870 C 1.2869354901 -2.5656851104 -1.0481346914 Si 1.3719988825 0.4484405685 -0.7886165828 H 0.6711931738 1.2422824414 -1.8145590778 H 1.2636299658 -3.6276960094 -1.1292809212

6 RCCSD(T)/CC-PV(T+D)Z,H=CC-PVTZ ENERGY=-655.26768139 C 1.9985312303 -1.1393459028 -1.3916410613 C 3.0736931554 -1.4736697577 -0.6944693013 Si 1.3998581549 -1.2897553804 0.3510846694 H 4.1872633871 -0.6896055521 1.3143162336 H 1.7335931229 -0.9368863635 -2.4167681278 Si 3.7914445793 -2.0898769334 0.8739323575

6 RCCSD(T)/CC-PV(T+D)Z,H=CC-PVTZ ENERGY=-655.21775340 C 1.7297709019 -1.3825517469 -1.5980483999 C 1.8293781884 -2.8122361416 -1.6473360593 Si 1.3710738448 0.0300896110 -0.6661939681 H 1.9790406775 -0.8471867903 -2.5442536731 H 2.1502511947 -3.2412022738 -2.6298590021 Si 1.6881234327 -4.3528627286 -0.8809385174

6 RCCSD(T)/CC-PV(T+D)Z,H=CC-PVTZ ENERGY=-655.19694965 C 1.9710668231 -1.6910843765 -1.4859215749 C 1.0678425994 -2.5755322435 -1.7345230315 Si 1.2742394367 -0.7927602354 0.0286291236 H 0.4082872287 0.3430879447 -0.4229520577 H 1.1175445272 -4.0328759270 0.6286293762 Si 0.3401189749 -2.9302055123 -0.0223560059

6 RCCSD(T)/CC-PV(T+D)Z,H=CC-PVTZ ENERGY=-906.31385554 C 1.0420670484 -1.2095193043 -0.9012184960 Si 1.4197810797 -2.7307812909 -1.9114013473 Si 2.0710542559 -0.8194507958 0.6051345550 H 0.3916925505 -0.4396073522 -1.3164997296 H -0.1694130620 -3.3383343167 1.0302301314 Si 1.0591198874 -2.8527342401 0.3434207967

6 RCCSD(T)/CC-PV(T+D)Z,H=CC-PVTZ ENERGY=-906.30748419 C 1.3756354498 -1.0506352985 -0.9904176800 Si 0.9395966050 -2.7748664340 -1.2888435800 Si 2.8303379355 -1.4430795267 0.0311226554 H 0.9003234851 -0.1124415708 -1.2539639438 H 0.8996277903 -3.4557458611 -2.6070429168 Si 1.0886819943 -2.8733527289 0.9286687451

6 RCCSD(T)/CC-PV(T+D)Z,H=CC-PVTZ ENERGY=-906.26571342 C 1.1461172406 -1.0422267086 -0.9410808013 Si 1.4951818082 -2.5152579426 -1.8283656474 Si 1.9891393082 -0.7952236528 0.5689039909 H 2.9019172144 0.3239006047 0.9133493414 H 0.0535147587 -2.9572792712 1.1845771908 Si 1.4447512198 -3.0204956895 0.6117538855

6 RCCSD(T)/CC-PV(T+D)Z,H=CC-PVTZ ENERGY=-1157.34810358 Si 2.0053221079 -1.3462029650 -0.7630315901 Si 2.1905506316 -3.6017131473 -0.6957208453 Si 2.0643239131 -1.5445987084 1.4463635011 H 0.9998265867 -1.3924830488 2.4722302949 H 2.4569573229 -0.2511357921 -1.6605560794 Si 3.7151374377 -3.0267520884 0.9944908186

Figure 6

Panel (b)

4 RCCSD(T)/VDZ ENERGY=-402.78628978 C 2.3173377849 -1.2988345314 -0.1445324654 C 1.0985298946 -1.2335174266 -1.0515243863 C 1.4240172269 -0.1433427110 -0.1356162323 Si 2.0969781536 -2.8053111310 -1.2317605960

Vibration Wavenumber Nr [1/cm] 1 232.89 2 399.97 3 657.01 4 941.35 5 1024.43 6 1377.31

Panel (c)

4 RCCSD(T)/VDZ ENERGY=-653.89671510 C 2.2594348797 -1.2855343777 -0.1680985977 C 1.0908914667 -1.2715517940 -1.0657532660 Si 2.1266838255 -2.8146683386 -1.2287136731 Si 1.2234915081 0.2574751802 -0.0052545132

Vibration Wavenumber Nr [1/cm] 1 187.80 2 351.55 3 490.28 4 936.85 5 960.99 6 1050.28

M06-2X

4 UKS-SCF000/AVTZ ENERGY=-655.03234122 C 2.2404941537 -1.2851862457 -0.1829470858 C 1.1104190987 -1.2716579182 -1.0514050034 Si 2.1193097633 -2.7896495537 -1.2182912837 Si 1.2302786642 0.2322143876 -0.0151766771

Panel (d)

4 RCCSD(T)/VDZ ENERGY=-653.77651533 C 2.3284585776 -1.3380698553 -0.1245805529 C 0.1790426252 -1.4986643592 -1.9499586351 Si 0.6562625866 -0.5183299288 -0.4129006505 Si 1.4296695507 -2.6497004267 -1.1360847515

Vibration Wavenumber Nr [1/cm] 1 298.99 2 425.05 3 525.89 4 544.38 5 646.72 6 705.32

M06-2X

4 UKS-SCF000/AVTZ ENERGY=-654.88784492 C 2.3089642599 -1.3532122334 -0.1012003397 C 0.1603154410 -1.5136354494 -1.9259855525 Si 0.6858263781 -0.5322290664 -0.4464193766 Si 1.4383272610 -2.6056878208 -1.1499193212

Vibration Wavenumber Nr [1/cm] 1 214.02 2 496.23 3 559.08 4 647.02 5 710.65 6 774.60

Panel (e), singlet

4 RCCSD(T)/VDZ ENERGY=-904.98436123 C 1.2097153592 -1.3030480505 -0.9086135842 Si 2.0072620006 -2.8205666657 -1.4340967392 Si 0.7222143991 0.1373158677 0.0416160047 Si 2.3685143011 -1.5914569514 0.6789022787

Vibration Wavenumber Nr [1/cm] 1 174.04 2 297.68 3 345.26 4 489.89 5 624.02 6 1073.51

M06-2X 4 UKS-SCF000/AVTZ ENERGY=-906.37826146 C 0.5576552735 -2.8670636055 -2.0158013960 Si 0.9852089452 -3.4376159837 -0.3260377973 Si 1.0623568456 -1.2760262913 -1.2573779422 Si 2.1301447156 -1.7129818195 0.7924415755

Vibration Wavenumber Nr [1/cm] 1 174.04 2 297.68 3 345.26 4 489.89 5 624.02 6 1073.51

Panel (f), singlet

4 RCCSD(T)/AUG-CC-PV(T+D)Z ENERGY=-1156.16206784 Si 1.9672577329 -2.6704994500 -0.8664141688 Si 1.1790586292 -0.5163685061 -0.0819987547 Si 3.0576926096 -1.5298387934 0.8428455420 Si 0.0886411282 -1.6570160207 -1.7912786684

Vibration Wavenumber Nr [1/cm] 1 68.76 2 248.15 3 344.20 4 432.57 5 470.26 6 505.08

TRIPLET

4 RCCSD(T)/VDZ ENERGY=-1156.00540489 Si 2.7708048651 -1.3208967029 0.1714982829 Si 0.6998054080 -1.2507580218 -1.3981445724 Si 2.2348136619 -2.9973102099 -1.3488544118 Si 1.2362005850 0.4259942547 0.1216919312

Vibration Wavenumber Nr [1/cm] 1 314.49 2 391.06 3 455.22 4 587.73 5 678.55 6 1706.32

4 RCCSD(T)/AUG-CC-PV(T+D)Z,H=AUG-CC-PVTZ ENERGY=-1156.13057036 Si 2.7521669624 -1.3202126473 0.1572560020 Si 0.7185576816 -1.2513402791 -1.3840483591 Si 2.2286735672 -2.9765101820 -1.3398238456 Si 1.2422263087 0.4050924285 0.1128074328

Vibration Wavenumber Nr [1/cm] 1 307.34 2 342.47 3 371.31 4 522.09 5 601.33 6 1568.36

Figure 7

Panel (a) M06-2X

10 UKS-SCF000/VDZ ENERGY=-407.26026866 C -2.4591903368 -0.5964809237 0.1429159998 Si -0.7830401346 -0.7568819653 -0.1389443433 C 1.5674064916 -2.2602936147 0.0281762390 H -2.9727905812 0.3376286941 -0.0941198041 H -0.2569751420 -3.1845675243 0.5264917029 C 0.2368489593 -2.2676747254 0.1888899931 H 2.1844281696 -3.1400847838 0.2307789670 H -3.0763314439 -1.3916275992 0.5671632786 H 0.0345592359 0.3585431916 -0.6809501974 H 2.0995455021 -1.3666380893 -0.3133935155

Vibration Wavenumber Nr [1/cm] 1 136.46 2 175.99 3 346.58 4 401.30 5 519.29 6 613.04 7 666.99 8 728.18 9 770.06 10 878.47 11 987.07 12 1012.75 13 1021.55 14 1118.50 15 1264.36 16 1401.26 17 1418.66 18 1684.43 19 2274.17 20 3150.42 21 3179.00 22 3187.55 23 3242.11 24 3277.45

Panel (b)

10 UKS-SCF000/VDZ ENERGY=-658.62698360 C -2.3493828946 -0.8399310605 0.0616691629 C -1.0159610837 -0.8426429683 -0.0814146127 Si 2.1738938799 -2.3183085942 0.0599057230 H -2.9496107993 0.0473116170 -0.1581071718 H

-0.6979840711 -3.4575324290 0.8617538307 Si 0.0294777830 -2.3521444560 0.1821543395 H 2.9550303642 -3.5757894279 0.1592817758 H -2.8971424724 -1.7243185318 0.4006467054 H -0.5172966447 0.0707817397 -0.4220361681 H 2.8504627086 -1.2343899889 -0.6975949445

Vibration Wavenumber Nr [1/cm] 1 103.90 2 124.20 3 191.65 4 302.64 5 348.65 6 460.58 7 520.12 8 537.45 9 583.18 10 729.50 11 757.64 12 912.93 13 995.76 14 1015.75 15 1115.92 16 1266.84 17 1427.78 18 1677.22 19 2255.39 20 2259.66 21 2286.79 22 3152.37 23 3183.35 24 3245.60

Panel (c) M06-2X

10 UKS-SCF000/VDZ ENERGY=-658.60663372 C -2.5701530144 -0.4581128925 0.0846633936 Si -0.8745149347 -0.4464737389 -0.1797909465 C 2.0725725818 -2.4363357834 -0.0026832287 H -3.2111709899 0.4102137649 -0.0847215002 H -0.3893593504 -3.4730979324 0.7984017350 Si 0.3787186044 -2.3266288055 0.2463316557 H 2.6274353460 -3.3444714925 0.2454408543 H -3.0789140694 -1.3574488976 0.4395957657 H -0.1587539656 0.7601180877 -0.6684623107 H 2.6643838322 -1.6191624498 -0.4217670983

Vibration Wavenumber Nr [1/cm] 1 81.41 2 108.17 3 221.41 4 311.95 5 355.08 6 448.68 7 578.27 8 645.85 9 679.41 10 680.79 11 767.93 12 816.04 13 865.03 14 872.42 15 982.88 16 993.95 17 1373.27 18 1398.02 19 2264.02 20 2265.25 21 3173.25 22 3177.46 23 3270.74 24 3274.24

Panel (d), M06-2X:

10 UKS-SCF000/VDZ ENERGY=-909.97941966 C -2.5881603449 -0.3867850719 0.0976153230 Si -0.9069268086 -0.5924673475 -0.1659913625 Si 2.4086553756 -2.3525476685 0.0981729641 H -3.0802419156 0.5593182412 -0.1402889588 H -0.3982800434 -3.7027306351 0.7802755338 Si 0.2708604452 -2.5209389568 0.1804002971 H 3.3554153490 -3.4754068964 0.3077246535 H -3.2197721233 -1.1700882679 0.5226486993 H -0.0758650356 0.5052909871 -0.7264791735 H 3.0231411817 -1.1392219943 -0.4970696561

Low Vibration Wavenumber Nr [1/cm] 1 0.00 2 0.00 3 0.00 4 0.00 5 0.00 6 0.00 7 15.29

Vibration Wavenumber Nr [1/cm] 1 99.48 2 161.94 3 214.73 4 301.16 5 383.33 6 425.39 7 488.89 8 545.16 9 599.32 10 620.81 11 664.38 12 712.53 13 771.65 14 884.27 15 926.50 16 992.50 17 1405.96 18 2259.70 19 2263.26 20 2268.98 21 2290.29 22 3175.30 23 3278.04

10 UKS-SCF000/CC-PV(T+D)Z,H=CC-PVTZ ENERGY=-910.04481566 C -2.5724425031 -0.3805705387 0.1047799614 Si -0.9092157060 -0.6015270490 -0.1807939644 Si 2.3935159350 -2.3620685808 0.0627361158 H -3.0776133759 0.5362847181 -0.1679617756 H -0.3903639143 -3.7137267592 0.7402102239 Si 0.2742561815 -2.4954292973 0.2547889033 H 3.3383628355 -3.4697207757 0.2641497215 H -3.1915199179 -1.1363479590 0.5679880980 H -0.0880553371 0.4587686610 -0.7891141572 H 3.0119018823 -1.1112400296 -0.3997748068

Imaginary Vibration Wavenumber Nr [1/cm] 1 19.24

Vibration Wavenumber Nr [1/cm] 1 60.05 2 102.88 3 174.63 4 303.18 5 394.33 6 420.19 7 487.85 8 537.76 9 604.30 10 625.22 11 660.01 12 691.80 13 778.56 14 872.42 15 926.79 16 1001.27 17 1397.89 18 2294.92 19 2298.53 20 2305.04 21 2323.08 22 3174.20 23 3264.76

Panel (d), M06-2X:

10 UKS-SCF000/VDZ ENERGY=-909.97905333 Si -2.5454478098 -0.8070692425 0.1111677965 C -0.8417411285 -0.8248585403 -0.1302982025 Si 2.3641387684 -2.3161583514 0.2074564344 H -3.4114104475 0.3503376205 -0.2209592326 H -0.5852472556 -3.4103938033 0.7516711109 Si 0.1885845174 -2.3206470928 0.0854967384 H 2.9985958047 -3.6614659760 0.1226309716 H -3.2706563151 -1.9848041723 0.6432729583 H -0.3624302164 0.0751255309 -0.5291120982 H 3.0471009223 -1.3270286329 -0.6750678169

Vibration Wavenumber Nr [1/cm] 1 79.99 2 94.95 3 195.34 4 260.09 5 281.74 6 361.55 7 469.65 8 513.87 9 525.02 10 536.69 11 605.22 12 738.01 13 778.12 14 824.07 15 917.96 16 929.42 17 956.12 18 1148.28 19 2223.01 20 2227.07 21 2251.40 22 2274.91 23 2300.61 24 3200.71

10 UKS-SCF000/CC-PV(T+D)Z,H=CC-PVTZ ENERGY=-910.04629686 Si -2.5261407708 -0.8301086031 0.0982259899 C -0.8329202957 -0.7782634699 -0.1037340053 Si 2.3578116485 -2.3271657816 0.1897607668 H -3.4371385574 0.2756602679 -0.2257169992 H -0.5881759989 -3.3222294597 0.7787200206 Si 0.1921635131 -2.2571275929 0.1113551049 H 2.9183461165 -3.6908045508 0.1096692542 H -3.1980522282 -2.0416209070 0.5818807527 H -0.3684729213 0.1286594081 -0.4723582722 H 3.0640663342 -1.3839619709 -0.7015439523

Vibration Wavenumber Nr [1/cm] 1 79.30 2 101.63 3 198.56 4 239.58 5 306.41 6 365.90 7 459.89 8 516.89 9 533.56 10 546.22 11 613.32 12 761.90 13 837.77 14 854.12 15 925.36 16 932.59 17 971.95 18 1144.41 19 2255.72 20 2259.54 21 2281.52 22 2314.26 23 2338.27 24 3197.63

Panel (f), M06-2X:

10 UKS-SCF000/VDZ ENERGY=-1161.35261126 Si -3.0601624274 -0.5150367989 0.0251403072 Si -0.8984707923 -0.4700657975 0.0247954358 Si 2.4278679887 -2.3324652842 0.1500130590 H -3.8654367777 0.7300043897 -0.0807275003 H -0.4128191322 -3.5518723712 0.8040624692 Si 0.2690969618 -2.4364182709 0.0900380960 H 3.2771776047 -3.5474816829 0.2604207759 H -3.7632688397 -1.6024261585 0.7555035229 H -0.2138075953 0.6219154866 -0.7224300001 H 3.1111564693 -1.2052200221 -0.5380883954

Low Vibration Wavenumber Nr [1/cm] 1 0.00 2 0.00 3 0.00 4 0.00 5 0.00 6 0.00 7 29.09

Vibration Wavenumber Nr [1/cm] 1 55.04 2 114.35 3 172.10 4 200.44 5 274.13 6 357.10 7 420.25 8 428.02 9 474.02 10 525.02 11 529.85 12 577.82 13 592.57 14 631.08 15 754.97 16 914.19 17 922.96 18 2242.08 19 2244.86 20 2246.40 21 2249.26 22 2274.89 23 2276.40

10 UKS-SCF000/CC-PV(T+D)Z,H=CC-PVTZ ENERGY=-1161.41757977 Si -3.0541913144 -0.5017125079 0.0570281687 Si -0.9070633027 -0.5015241772 -0.0175130999 Si 2.4189264876 -2.3302815255 0.1451760702 H -3.8454141658 0.7323474388 -0.0775624684 H -0.4056239427 -3.5692027525 0.7608521336 Si 0.2734362207 -2.4413181041 0.0973900462 H 3.2795008745 -3.5195559183 0.2624860318 H -3.7653019232 -1.5707971346 0.7769860218 H -0.2170025849 0.5786475276 -0.7479310128 H 3.0940671110 -1.1856693564 -0.4881841214

Low Vibration Wavenumber Nr [1/cm] 1 0.00 2 0.00 3 0.00 4 0.00 5 0.00 6 0.00 7 40.11

Vibration Wavenumber Nr [1/cm] 1 53.67 2 109.91 3 163.20 4 220.94 5 323.83 6 380.76 7 430.72 8 450.09 9 493.42 10 532.42 11 547.21 12 584.57 13 599.34 14 632.13 15 756.12 16 923.78 17 932.89 18 2278.99 19 2283.26 20 2285.71 21 2288.85 22 2310.73 23 2313.17

Figure 13

Panel (a)

20 UKS-SCF000/USERDEF ENERGY=-2259.10608880 C 0.2430113450 2.2256731775 0.2112321005 Si -1.3592656971 1.3979455427 0.0616775383 Si 1.8365452158 1.4614972109 0.1745439479 Si -2.6205075481 -1.4235686000 -0.0850687223 C -1.1654684946 -0.4478707282 -0.0367405909 Si 0.3893331373 -1.2849034262 0.0026360912 C 1.9061237899 -0.3631645331 0.0619344646 Si 3.4273681276 -1.2345123540 0.0798066761 C -2.6142614831 -3.1887870417 -0.0756049044 Si -1.0685633844 -3.9880208793 -0.0361595607 C 0.4571510205 -3.0926641472 -0.0057692361 Si 2.0325482588 -3.8881964438 0.0246178020 C 3.5285608564 -2.9927290102 0.0528256144 H 0.2011703063 3.3077607052 0.3007386937 H 4.6836661129 -0.4599154424 0.1245235501 H -3.9293671480 -0.7426014282 -0.1285697908 H 2.0510405337 -5.3655886593 0.0252989577 H -0.9856647507 -5.4635948294 -0.0261183814 H 4.4843199075 -3.5031887957 0.0645691650 H -3.5390364055 -3.7534888376 -0.0906895851

Vibration Wavenumber Nr [1/cm] 1 71.54 2 74.57 3 92.45 4 109.48 5 130.60 6 181.56 7 194.69 8 196.41 9 221.32 10 223.28 11 228.60 12 244.42 13 270.43 14 272.41 15 360.14 16 393.48 17 401.14 18 401.70 19 423.44 20 536.04 21 538.63 22 541.64 23 559.14 24 580.10 25 598.06 26 694.49 27 705.40 28 723.96 29 745.90 30 755.02 31 757.21 32 780.14 33 792.91 34 845.79 35 880.40 36 886.32 37 897.84 38 918.95 39 951.11 40 960.39 41 987.61 42 1018.70 43 1042.60 44 1093.15 45 1107.83 46 1133.35 47 1136.81 48 2287.78 49 2292.83 50 2294.95 51 2296.93 52 3189.12 53 3206.33 54 3209.80

Panel (b)

20 UKS-SCF000/USERDEF ENERGY=-2259.10058861 C 0.5689598230 2.5471540825 0.1044080153 Si -0.8861050494 1.5446836902 0.0157390717 Si 2.1079950113 1.7368868717 0.0635826413 Si -2.5796125284 -1.0346967694 -0.0895262648 C -0.8728607084 -0.2069830415 -0.0413639033 Si 0.7059203957 -0.9915073420 -0.0123342587 C 2.2422235478 -0.0322745126 0.0041936876 Si 3.7965911744 -0.8897863988 0.0114817225 C -2.2521958545 -2.8414185286 -0.0588491245 Si -0.7291627139 -3.6456589533 -0.0020537415 C 0.8336937251 -2.7703892750 0.0082520384 Si 2.4017093540 -3.5451177888 0.0366873013 C 3.9094929346 -2.6445730524 0.0231472110 H 0.4926211261 3.6278754018 0.1650468337 H 4.8661591950 -3.1525631231 0.0314069073 H -3.1410372046 -3.4697849608 -0.0718001879 H 3.3731799482 2.5002945395 0.0819617233 H -2.1851056789 2.2448622420 -0.0051674243 H 2.4572151861 -5.0216832760 0.0728171285 H -0.5753509134 -5.1213551352 0.0407826333

Low Vibration Wavenumber Nr [1/cm] 1 0.00 2 0.00 3 0.00 4 0.00 5 0.00 6 0.00 7 37.93

Vibration Wavenumber Nr [1/cm] 1 76.64 2 100.40 3 126.40 4 162.44 5 195.24 6 197.92 7 211.01 8 222.27 9 225.31 10 236.26 11 252.79 12 268.84 13 278.55 14 358.07 15 388.42 16 401.64 17 415.40 18 418.28 19 505.02 20 520.93 21 532.19 22 554.37 23 560.10 24 586.10 25 639.49 26 658.48 27 706.64 28 730.00 29 750.91 30 761.34 31 831.99 32 845.13 33 858.24 34 875.67 35 918.13 36 929.51 37 947.52 38 985.28 39 1003.20 40 1023.05 41 1045.43 42 1088.85 43 1105.18 44 1120.99 45 1132.98 46 1163.78 47 2247.77 48 2284.60 49 2289.90 50 2298.87 51 3150.66 52 3205.83 53 3213.56

Panel (c)

20 UKS-SCF000/USERDEF ENERGY=-2259.10030885 C 0.2851942154 2.3120099430 0.0984178630 Si -1.1844415705 1.3503930227 0.0431226655 Si 1.8041813388 1.4495248786 0.1285889325 Si -2.6343406735 -1.4386108161 -0.0224780226 C -1.1718793880 -0.4005129741 0.0150551749 Si 0.3795346995 -1.2845748185 0.0401948025 C 1.8992523450 -0.3112159013 0.1022467429 Si 3.4386768528 -1.1885164807 0.1241966947 C -2.5615315967 -3.1606444549 -0.0258902907 Si -1.0748193723 -4.2414572326 -0.0303521373 C 0.4007007669 -3.0480022556 0.0062321194 Si 1.9858208267 -3.8136157997 0.0204066898 C 3.5281439430 -2.9250924833 0.0753893916 H 0.2532550832 3.3960176651 0.1132954802 H 4.6658527216 -0.3647695147 0.1863388601 H -3.9015025933 -0.6667450932 -0.0487233071 H 3.0742291572 2.2023699925 0.1754180269 H -2.4895954698 2.0408061662 0.0205451362 H 4.4783592440 -3.4453002771 0.0831492536 H -3.5103659400 -3.6929971765 -0.0400013960

Vibration Wavenumber Nr [1/cm] 1 68.93 2 81.09 3 109.67 4 132.09 5 174.29 6 178.90 7 195.15 8 215.26 9 224.02 10 228.40 11 237.51 12 253.06 13 271.69 14 282.07 15 356.19 16 392.43 17 399.96 18 411.83 19 422.75 20 530.22 21 539.29 22 550.03 23 555.29 24 564.06 25 596.52 26 640.19 27 698.23 28 712.47 29 731.41 30 751.82 31 794.79 32 815.16 33 843.45 34 852.76 35 893.07 36 906.17 37 917.70 38 956.77 39 982.87 40 1010.15 41 1021.62 42 1037.55 43 1084.42 44 1096.95 45 1126.69 46 1133.36 47 1165.20 48 2245.00 49 2271.56 50 2289.87 51 2293.65 52 3159.08 53 3211.82 54 3213.98

Panel (d)

20 UKS-SCF000/USERDEF ENERGY=-2259.01428632 C 0.5503961976 2.6026800849 0.0364027337 Si -0.9167197935 1.6610834385 0.0151668999 Si 2.0780198593 1.7232159295 0.0610398566 Si -2.3989711102 -1.0879184272 -0.0152282153 C -0.9185240426 -0.1090695810 0.0126289932 Si 0.6679650950 -0.9703414280 0.0274102772 C 2.1712224025 -0.0245325471 0.0579183783 Si 3.7326373630 -0.9785912874 0.0790376832 C -2.2522089228 -2.8146127689 -0.0276454168 Si -0.7300380316 -3.6788450878 -0.0188573323 C 0.7583270958 -2.7477450924 0.0067551954 Si 2.4409313941 -3.4735623846 -0.0070262893 C 3.9660858183 -2.6826921428 0.0473055139 H 0.5225768296 3.6871283603 0.0353423531 H 4.8537781293 -0.0043283480 0.1242879349 H -3.7075687165 -0.4093774106 -0.0271143968 H 3.3324088102 2.5005186235 0.0830353481 H -2.2340706656 2.3285177560 -0.0036315711 H 2.2852687692 -4.9496549894 -0.0785336152 H -0.6987456510 -5.1521282774 -0.0298823407

Vibration Wavenumber Nr [1/cm] 1 67.67 2 76.00 3 89.47 4 124.72 5 183.39 6 190.85 7 193.88 8 199.34 9 216.72 10 221.93 11 238.72 12 254.63 13 266.33 14 283.01 15 361.35 16 379.42 17 390.16 18 395.48 19 416.87 20 527.94 21 539.76 22 545.32 23 554.96 24 560.29 25 574.20 26 596.38 27 602.79 28 685.51 29 715.88 30 720.44 31 748.31 32 767.55 33 782.83 34 839.61 35 855.85 36 890.26 37 924.20 38 939.77 39 970.21 40 991.03 41 1017.55 42 1047.61 43 1096.50 44 1114.70 45 1123.45 46 1144.76 47 1170.70 48 2230.75 49 2235.73 50 2289.91 51 2298.17 52 2306.65 53 2317.24 54 3211.30

Panel (e)

20 UKS-SCF000/USERDEF ENERGY=-2007.64333294 Si 0.3397867189 2.7157528971 0.0500527923 C -0.4062325389 1.1724799878 0.0648296341 C 1.1625488426 1.2112963511 0.0565737285 C -2.6063400321 -1.2952089599 0.0540609299 Si -1.0358759711 -0.5347663334 0.0552313456 C 0.4438924266 -1.4991833967 0.0405566146 Si 1.8753375941 -0.4634827885 0.0435691002 C 3.4807597891 -1.1485200086 0.0322457833 Si -2.5496600709 -3.0644210959 0.0139569955 C -1.1078952815 -4.0561043949 -0.0295078175 Si 0.4869295359 -3.2846826526 0.0065158602 C 2.1168573461 -3.9793215121 0.0123569121 Si 3.5101639772 -2.9190903969 0.0223320389 H 0.3001684349 4.1845571010 0.0331119917 H 4.4025271035 -0.5811069439 0.0318733319 H -3.5541210856 -0.7732272495 0.0693901374 H 2.2871438600 -5.0515743471 0.0079681600 H -1.2316089100 -5.1340914560 -0.0709373746 H -3.8573758987 -3.7546813349 0.0161378512 H 4.8503024300 -3.5447283062 0.0208026845

Vibration Wavenumber Nr [1/cm] 1 69.14 2 103.78 3 117.07 4 175.34 5 184.24 6 206.08 7 224.19 8 233.94 9 251.23 10 268.58 11 273.75 12 318.46 13 346.55 14 355.41 15 367.53 16 396.17 17 399.89 18 463.11 19 464.17 20 498.53 21 552.54 22 559.15 23 572.74 24 614.83 25 699.44 26 714.33 27 736.45 28 743.99 29 751.46 30 759.83 31 776.19 32 779.44 33 825.89 34 850.12 35 874.55 36 896.77 37 933.75 38 937.61 39 961.36 40 992.92 41 1052.55 42 1068.65 43 1076.42 44 1117.89 45 1128.71 46 1156.30 47 1282.55 48 2278.04 49 2279.54 50 2329.68 51 3193.66 52 3194.01 53 3218.98 54 3224.36
